# Visible Light Control over the Cytolytic Activity of a Toxic Pore-Forming Protein

**DOI:** 10.1021/acschembio.3c00640

**Published:** 2024-02-06

**Authors:** Jana Volarić, Nieck J. van der Heide, Natalie L. Mutter, Douwe F. Samplonius, Wijnand Helfrich, Giovanni Maglia, Wiktor Szymanski, Ben L. Feringa

**Affiliations:** † Stratingh Institute for Organic Chemistry, University of Groningen, 9747 AG Groningen, The Netherlands; ‡ Groningen Biomolecular Sciences and Biotechnology Institute, University of Groningen, 9747 AG Groningen, The Netherlands; § Department of Surgery, Translational Surgical Oncology, University of Groningen, University Medical Center Groningen, Hanzeplein 1, 9713 GZ Groningen, The Netherlands; ∥ Department of Radiology, Medical Imaging Center, University of Groningen, University Medical Center Groningen, 9713 GZ Groningen, The Netherlands

## Abstract

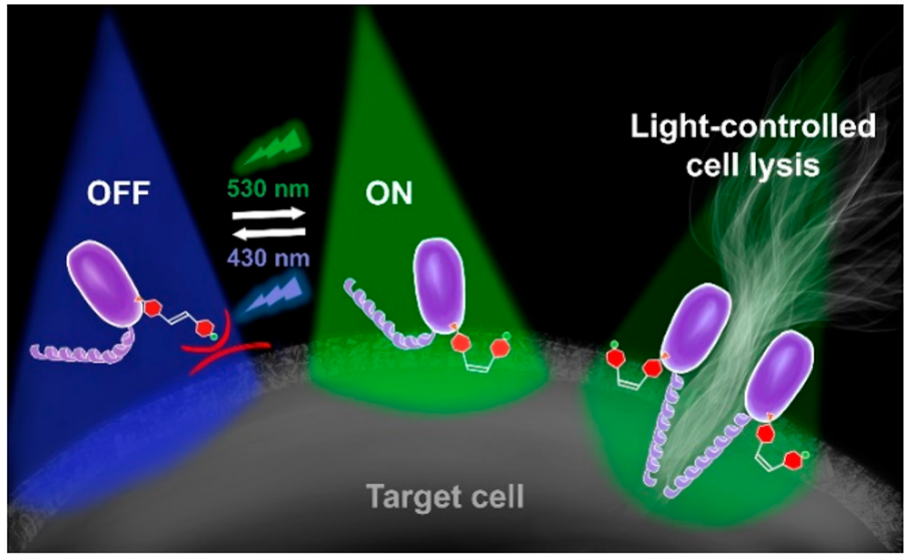

Enabling control over the bioactivity of proteins with light, along with the principles of photopharmacology, has the potential to generate safe and targeted medical treatments. Installing light sensitivity in a protein can be achieved through its covalent modification with a molecular photoswitch. The general challenge in this approach is the need for the use of low energy visible light for the regulation of bioactivity. In this study, we report visible light control over the cytolytic activity of a protein. A water-soluble visible-light-operated tetra-*ortho*-fluoro-azobenzene photoswitch was synthesized by utilizing the nucleophilic aromatic substitution reaction for installing a solubilizing sulfonate group onto the electron-poor photoswitch structure. The azobenzene was attached to two cysteine mutants of the pore-forming protein fragaceatoxin C (FraC), and their respective activities were evaluated on red blood cells. For both mutants, the green-light-irradiated sample, containing predominantly the *cis*-azobenzene isomer, was more active compared to the blue-light-irradiated sample. Ultimately, the same modulation of the cytolytic activity pattern was observed toward a hypopharyngeal squamous cell carcinoma. These results constitute the first case of using low energy visible light to control the biological activity of a toxic protein.

## Introduction

Toxins are poisonous substances produced by living organisms, which are present in all kingdoms of life.^1−6^ A number of known protein-based toxins belong to the pore-forming toxins (PFTs) family.^7,8^ PFTs are usually produced as water-soluble monomers, with the ability to bind to target membranes and assemble into an oligomeric pore complex, thereby disrupting or perforating the lipid bilayer.^7,8^ Typically, organisms employ PFTs to gain entry into host cells to acquire nutrition or use PFTs as a defense mechanism.^9−11^ Of note, the human immune system involves pore-forming proteins in its defense machinery.^12,13^ Consequently, PFTs are known to play a crucial role in the virulence mechanism of various pathogens.^14−17^

While some of these inherently toxic proteins have targeting capabilities, such as the latrotoxin of the deadly black widow spider that selectively punctures neurons,^18,19^ others, like actinoporins, lyse indiscriminately all sphingomyelin-containing membranes.^20−22^ Since all mammalian cell surfaces contain sphingomyelin, the cytolytic activity of actinoporin PFTs can potentially result in the destruction of human cells. However, to precisely and safely utilize PFTs as novel chemotherapeutics, it is necessary to establish external control over their pore-formation ability. Under physiological conditions, the pore formation of PFTs is triggered by the lipid composition of the target cell membrane,^23−25^ the presence of specific cell surface receptors,^26,27^ proteolysis,^28^ or pH changes in the intracellular microenvironment.^29^ Several PFTs with nonspecific cytolytic activity have been modified with antibody fragments for targeting toward specific cell lines,^30−32^ with some requiring additional protease activation to initiate the pore-forming process.^30,33,34^ However, none of the aforementioned strategies allow for external control without substantial modification of the PFT by incorporating additional protein domains.^35^ Taken together, to allow for future therapeutic application, it is key to establish external, spatiotemporal control over the nonspecific cytolytic activity of PFTs.

In this context, light is an excellent stimulus that can be easily externally delivered at specific time points and locations.^36−39^ Organic chemistry provides artificial actuators that can reversibly respond to different wavelengths of light: molecular photoswitches.^39−42^ Photoswitches are small organic molecules that change their shape and properties in a reversible manner upon irradiation with light.^40^ They have been applied, *inter alia*, in photopharmacological approaches to control the action of small molecules, DNA, peptides, and, most importantly in this context, proteins.^36,38,41−47^ The structure and activity of numerous proteins have been manipulated by light with the help of covalently attached photoswitches via single attachment or two-point attached cross-linkers.^42,44,48^

To the best of our knowledge, there are only two reported examples of using light for external control over the activity of PFTs. In 1995, the Bayley group reported a photocaged β-hemolysin PFT whose cytolytic activity could be irreversibly triggered by UV-light irradiation.^49^ More recently, our group has published the first cell-lysing fragaceatoxin C (FraC) construct which reversibly performed controlled nanopore assembly with application of UV and white light.^50^ FraC labeled with a water-soluble azobenzene at strategic locations lysed both blood and cancer cells upon light activation (*cis* isomer), while the inactivated *trans* isomer did not show cytotoxicity at the same concentrations. However, UV-light irradiation was required for both of the described systems, which effectively prohibited their use in a biological system. UV light is not biocompatible as it can damage tissue and mutate DNA and has a low penetration depth.^51−56^ Therefore, it is necessary to develop a PFT-based system that can be fully operated with visible light, thus allowing external spatiotemporal manipulation, to bring this concept closer to a photocontrolled PFT-based therapeutic approach.

The major challenge of shifting the absorbance of photoswitches, in particular the most often applied azobenzenes, to the visible light region, has attracted much attention in the recent years.^57−63^ Visible-light-responsive and water-soluble systems often feature many drawbacks, such as difficult synthesis, insolubility in water, susceptibility to glutathione reduction, and short half-lives.^58,65−67^ Therefore, there are only a few examples of fully visible-light-controlled manipulation of proteins. In the majority of cases, freely diffused photoswitches, namely tetra-*ortho*-fluoro substituted^68−75^ and tetra-*ortho*-chloro substituted azobenzene,^61,76^ diazocines,^77^ or other photoswitches,^78^ were used to modulate the activity of proteins. However, there are only a few examples in which the photoswitch is covalently attached to a protein. As such, tetra-*ortho*-fluorinated azobenzene-based amino acids were encoded into mammalian cells via genetic code expansion enabling incorporation of the switch *in vivo*.^79,80^ Furthermore, both fluorinated and chlorinated azobenzenes were tethered to ionotropic receptors to modulate their activity.^81,82^ Visible light-responsive photoswitches with two attachment points, so-called cross-linkers, have been introduced as a staple to control the secondary structure of bioactive short peptides.^58,75,83−85^ However, to the best of our knowledge, there is only one example in which the visible-light responsive switch (based on the tetra-*ortho*-methoxy azobenzene core) was utilized to control a protein function through cross-linking the binding site of a guanine-N7 methyltransferase to achieve visible-light mediated methylation of the 5′ cap of mRNA.^86^ Nevertheless, none of the described examples exhibited pharmacological (cytolytic or cytotoxic) activity, specifically, when targeting cancer cells. Therefore, in this study, we report for the first time the fully visible-light-mediated control of protein toxicity with a water-soluble molecular photoswitch.

## Results and Disscussion

### Molecular Design

**Figure 1:**
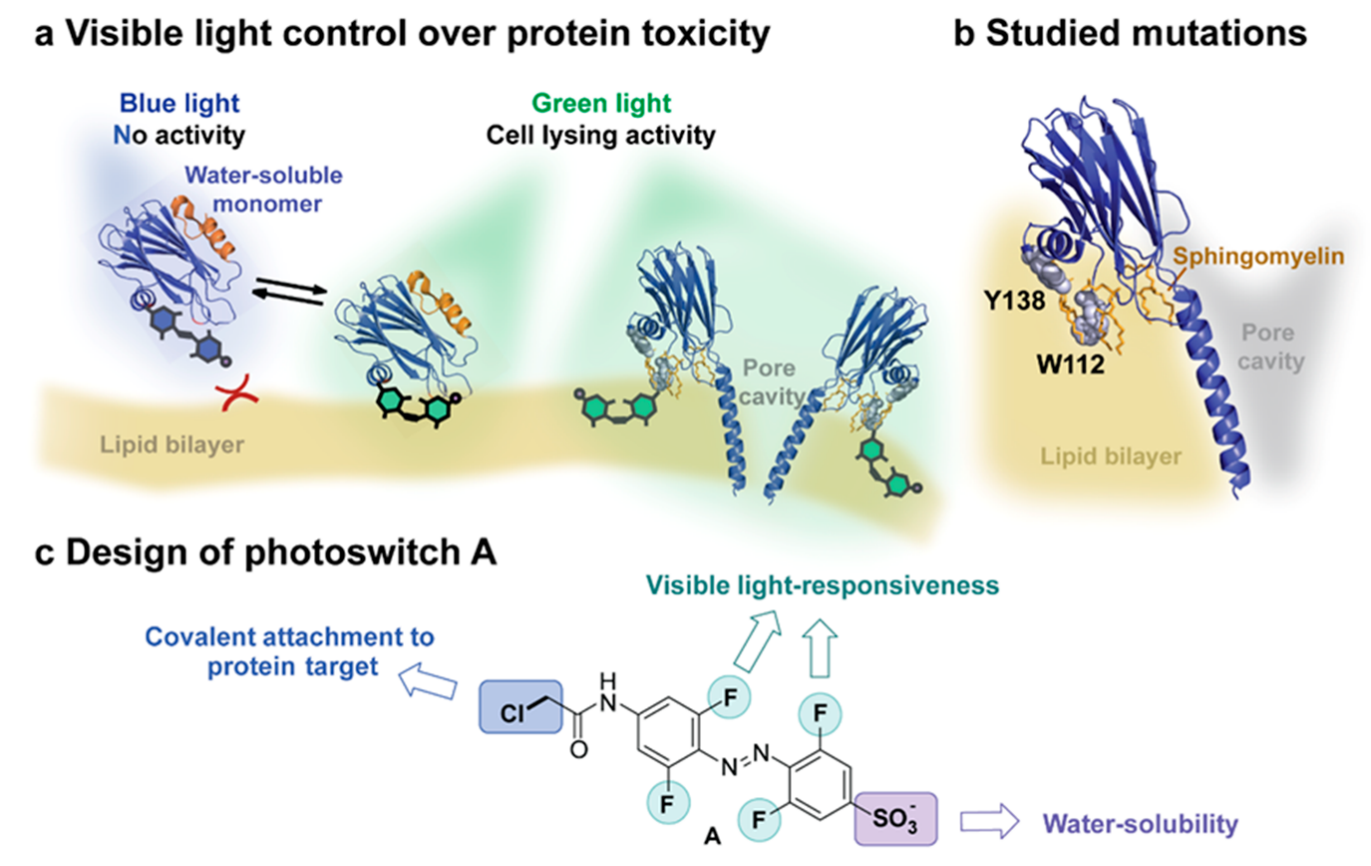
(a) Schematic representation of a FraC monomer (PDB 4TSY(64)) labeled with an azobenzene photoswitch; the *trans* isomer inhibits binding of the monomer to the lipid bilayer, while upon irradiation with green light the formed *cis* isomer allows binding to the membrane and further assembly into the oligomeric pore complex in the target membrane (yellow), (b) Schematic representation of a FraC monomer, inserted into the target membrane, where the residues that were herein mutated into cysteine are highlighted (gray spheres), as well as sphingomyelin molecules stabilizing the pore (orange). (c) Design elements of a visible-light-responsive photoswitch **A** with a water-solubilizing group and an attachment handle for covalent protein modification.

The ability to be operated with light that features low toxicity and deep tissue penetration is crucial for advancing the light-responsive PFT design toward developing a potential therapeutic. Therefore, we aimed to design a photoswitchable pendant for covalent attachment to the PFT that can be fully controlled with visible light (Figure 1c). Due to its synthetic accessibility and generally favorable photophysical properties,^41,48,55^ the azobenzene photoswitch was chosen. It has been shown in literature that introduction of four substituents in the *ortho* positions of the azobenzene core results in n−π* band separation for the two isomers allowing manipulation by visible light.^56−58,62,87^ Specifically, we selected the tetra-fluorinated core for three reasons: first, due to its optimal photophysical properties, such as generally long half-lives of the metastable *cis* state and high photostationary state distribution (PSD) ratios;^87−90^ second, known stability toward glutathione reduction; and third, because of the electron-poor nature of the *ortho*-fluoro azobenzene, which allows for later stage functionalization via nucleophilic aromatic substitution (S_N_Ar).^91^

Next to visible light, the issue of water solubility of inherently aromatic photoswitches poses a great challenge for their applications in biological systems.^42^ The main limitations for the introduction of water-solubilizing groups into the photoswitch structure include challenging synthesis and purification and the often detrimental effects of the solubilizing group and the aqueous medium on the photophysical properties of the switch. Therefore, the possibility to modify the electron-poor fluorinated azobenzene via S_N_Ar to introduce a water-solubilizing group at a later stage in the synthetic route, instead of starting with water-soluble compounds, would enable working with standard organic solvents during most of the synthetic route and minimizes repeated tedious purification under aqueous conditions. Furthermore, utilizing S_N_Ar to functionalize the azobenzene core provides an orthogonal and selective method that accommodates other functionalization approaches, such as cross-coupling reactions.

The PFT chosen for photoswitch modification was FraC, a relatively small protein (21 kDa) with a known crystal structure that is relatively easy to purify.^64,92−94^ FraC monomers assemble into octameric pores upon encountering cell membranes containing sphingomyelin, a lipid which is present in all mammalian cell surfaces.^64,95^ Since wild-type FraC does not contain a cysteine residue, the visible-light-responsive photoswitch furnished with a reactive chloroacetyl group was attached to a free cysteine genetically introduced by site-specific mutation, providing an extra level of precision in the design. The mutation sites, namely, W112 and Y138, were selected for their location at the interface of the formed oligomeric pore and the lipid bilayer of the target cell (Figure 1a).^64^ Furthermore, the selected positions are located in the sphingomyelin binding pockets. Sphingomyelin naturally stabilizes the assembled wild-type FraC pore,^64^ therefore we anticipated that modification of FraC at the selected locations would substitute sphingomyelin with the photoswitch and thus have a larger effect on the nanopore assembly process. After purification of the labeled FraC mutants, the hemolytic activity was evaluated using defibrinated red blood cells and human hypopharyngeal squamous cell carcinoma.

### Synthesis

**Scheme 1:**
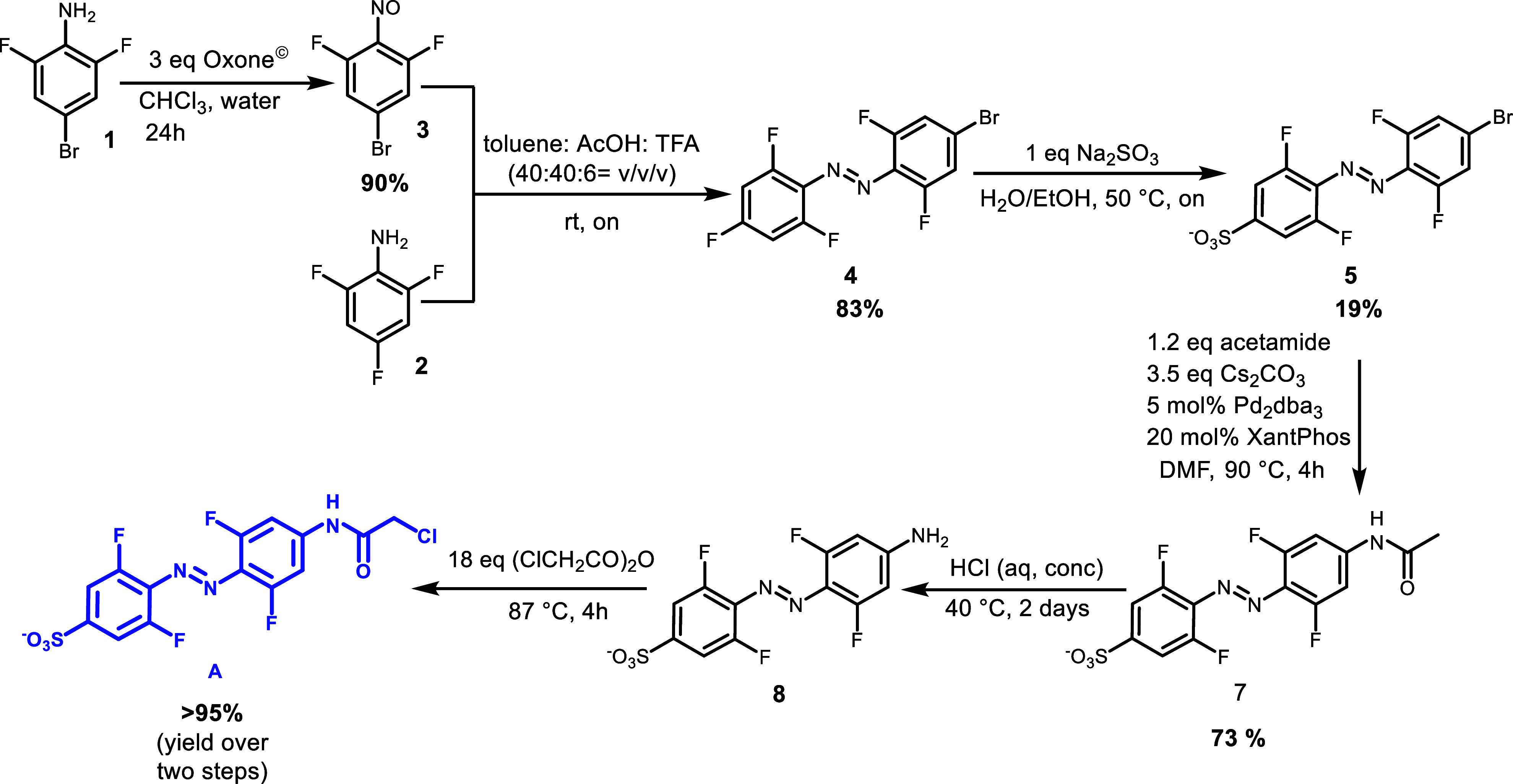
Synthesis of Water-Soluble Visible Light-Responsive Switch **A**

For the synthesis of the water-soluble visible-light-responsive switch **A**, we utilized the unique susceptibility of the tetra-*ortho*-fluoro-azobenzene core to S_N_Ar for installing the water solubilizing group (Scheme 1). Aniline **1** was oxidized to the nitroso derivative **3** and subsequently submitted to the Baeyer–Mills reaction with aniline **2** in a mixture of solvents (toluene, acetic acid, and TFA) to yield azobenzene **4** in good yield (83%) (Scheme 1). The sulfonation step was performed with azobenzene **4** and a weak nucleophile, sodium sulfite, in a 1/1 mixture of water and EtOH to ensure solubilization of both substrates. The water-soluble azobenzene **5** was purified by reverse phase chromatography using a mildly basic ammonium bicarbonate buffer and ACN mixture as eluents. After isolation of the pure sulfonated product **5**, Buchwald–Hartwig coupling with acetamide was performed at 90 °C in degassed DMF. Pure azobenzene **7** was isolated after reversed phase purification in a high yield (73%). The acetamide was hydrolyzed in the presence of concentrated hydrochloric acid and the isolated amine **8** underwent reaction with chloroacetic anhydride (neat) to obtain final photoswitch **A**. Azobenzene **A** was purified from the excess anhydride by several centrifuge washes with ether and fully characterized by NMR spectroscopy, HRMS, and LCMS analysis (See Supporting Information sections 1.2 and 3.1).

### Photophysical Properties

**Figure 2:**
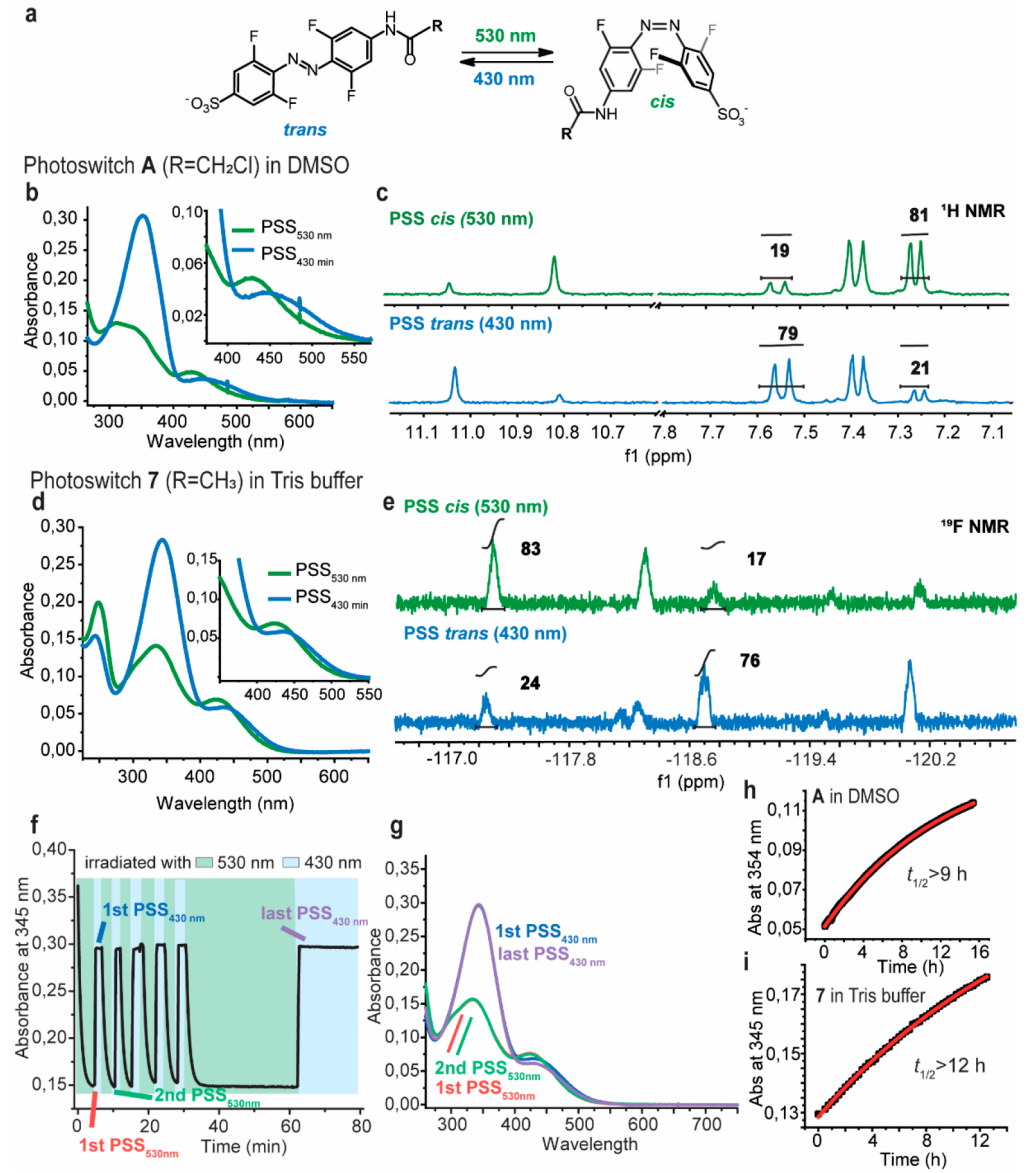
(a) Scheme depicting photoswitching of **A** (R = CH_2_Cl) and **7** (R = CH_3_) with visible light. (b) UV–vis spectrum of A at PSS_530nm_ and PSS_430nm_ (80 μM, DMSO). (c) PSD determination of **A** with ^1^H NMR spectroscopy (2 mM, DMSO-*d*_6_) by relative integration of the shifted signals shown in bold. (d) UV–vis spectrum of **7** at PSS_530nm_ and PSS_430nm_ (50 μM, 15 mM Tris, 150 mM NaCl, pH = 7.5). (e) PSD determination of **7** in Tris buffer with ^19^F NMR spectroscopy (0.88 mM, 15 mM Tris, 150 mM NaCl, pH = 7.5 with 10% DMSO-*d*_6_) by relative integration of the shifted signals shown in bold. The signal between −117.8 and 118.6 in the ^19^F-NMR is likely due to a fluorinated impurity which was impossible to be removed, despite several purification attempts. The signal does not react to irradiation. (f) Stability of **7** (50 μM) toward reduction by GSH (10 mM) in 15 mM Tris buffer, 150 mM NaCl, pH = 7.5, at 20 °C. Fatigue resistance in the presence of GSH with marked time points at which the UV–vis spectra are shown in panel g.(h) Half-life determination of **A** at 80 μM in DMSO. (i) Half-life determination of **7** at 50 μM in 15 mM Tris, 150 mM NaCl, pH = 7.5. All at 20 °C.

First, the photophysical properties of **A** were investigated in DMSO as a standard solvent for validating the photophysical properties of switches for biological applications. The UV–vis spectrum of azobenzene **A** clearly shows separation of the n−π* bands of both isomers thus allowing efficient switching exclusively with visible light (Figure 2b,c,h). The band separation is sufficient to ensure good isomer distribution at the photostationary state (PSS), as determined by NMR spectroscopy, revealing that high amounts of the respective isomer were formed upon irradiation with green (530 nm) and blue (430 nm) light (PSD_530nm_ = 81% *cis*, PSD_430nm_ = 79% *trans*). The photophysical properties in DMSO, including fatigue resistance over multiple switching cycles, were accompanied by a half-life over 9 h at 20 °C.

Furthermore, we tested the photophysical properties in an aqueous environment as similar as possible to the buffer used to elute the FraC monomer. This is important, since many azobenzenes show a substantially shorter half-life of the metastable *cis* isomer in water.^42,96^ While photoswitch **A** completely dissolved in Tris buffer at a concentration of 10 mM, it also contains a reactive chloroacetyl group highly susceptible to reaction with nucleophiles, such as Tris, which is the main component of the buffer used for the purification of FraC. Therefore, analogue **7** not bearing the chloroacetyl moiety (Figure 2d,e,i) was used as a model compound for studying the photophysical properties in Tris buffer. To our delight, azobenzene **7** also exhibited favorable photophysical properties in the aqueous medium with similar PSS ratios (PSD_530nm_ = 83% *cis*, PSD_430nm_= 76% *trans*), showing no fatigue upon numerous photoswitching cycles and, most importantly, a half-life of over 12 h at 20 °C that is sufficient for the PFT activity assays.

Furthermore, azobenzene **7** was used to assess the stability of the switch in the presence of glutathione (GSH). Resistance to GSH reduction is often tested for photoswitches used in biological applications since the environment in living cells is naturally highly reducing.^97−99^ Azobenzene **7** underwent several switching cycles in the presence of 10 mM GSH solution in Tris buffer indicating resistance to GSH reduction (Figure 2f,g). After investigating the photophysical properties of the water-soluble, visible-light-responsive molecular photoswitches in both DMSO and Tris buffer, it was concluded that molecule **A** can be reversibly and fully controlled with visible light while remaining stable in the presence of GSH and having a half-life sufficiently long for the cell lysis assays used to assess the reactivity of PFTs.

### Protein Labeling, PFT Activity Assays, and Cell Lysis

Two mutants of FraC with cysteine located at positions 138 or 112 were chosen for attachment of visible-light-responsive molecule **A** (Figure 1b). In the wild type both cysteines are located at the interface of the formed pore and the lipid bilayer, in the locations where naturally the sphingomyelin binding pocket is positioned.^64^ Sphingomyelin stabilizes the formation of the full pore complex and, in the wild-type pore, its presence in the target membrane is crucial for nanopore assembly.^64^ Therefore, we anticipated that introduction of an aromatic photoswitch in the sphingomyelin pocket locations could have the largest effect on the pore-forming activity.

Covalent attachment of switch **A** to the FraC monomers containing a freely accessible cysteine was performed in a degassed phosphate buffer solution in order to prevent oxidation of the free thiol. The presence of a mild reducing agent, such as tris(2-carboxyethyl)phosphine (TCEP), was avoided during the labeling reaction since the reactive chloroacetyl group of switch **A** can react with the reagents.

After labeling, the excess azobenzene was removed via size exclusion chromatography, while the content of any remaining unmodified monomer is the same across samples, possibly causing some background activity. For details of protein expression, labeling, and purification, see SI 1.4.

**Figure 3:**
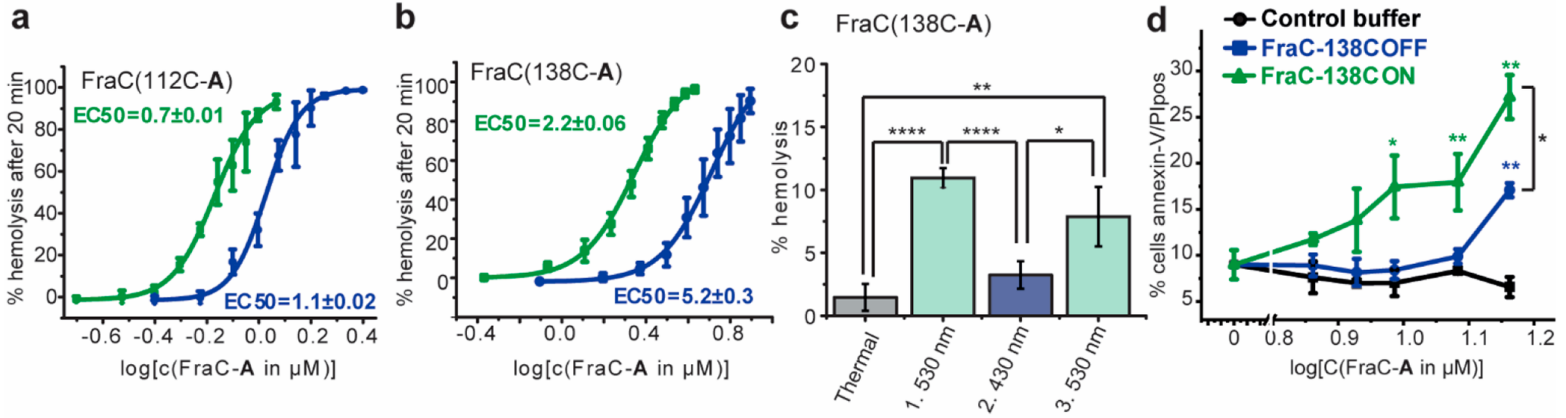
Hemolytic activity of labeled FraC–**A** upon irradiation with 530 (in green) and 430 (in blue) nm LED where the EC50 values are expressed in μM: (a) EC50 curves for FraC(W112C)–**A**; (b) EC50 curves for FraC(Y138C)–**A**. (c) Reversibility test of hemolytic activity of mutant FraC(Y138C) where the *P* values were determined by one-way ANOVA analysis between the highlighted conditions, **A** after 10 min upon subsequent irradiation cycles (approximately 2 μM). (d) Cytolytic activity of FraC(Y138C)–**A** on FaDu cancer cells where the *P* values were determined by one-way ANOVA analysis between the ON (green) or OFF (blue) and the control and between ON and OFF (highlighted with bracket) conditions. The asterisks represent *P* values as follows: ****P ≤ 0.0001; ***P ≤ 0.001; **P ≤ 0.01, and *P ≤ 0.05. Experiments a, b and c were performed as four independent replicates, while d was performed in duplicate.

The cytolytic activity of the labeled FraC constructs was evaluated by performing a hemolytic activity assay on defibrinated red blood cells. In the initial studies, it was observed that ambient light, even stray light from computer screens, significantly affected the hemolytic activity of the constructs. This observation was in accordance with the finding that the free switch **A** and its derivatives, being responsive to visible light, very efficiently formed a mixture of both isomers under any ambient light. Therefore, all the hemolytic activity assays, as well as handling of the samples after labeling with switch **A**, were performed strictly under dim red light. Before addition to the red blood cells, the freshly prepared labeled FraC mutants were preirradiated with either green (530 nm, “ON”) or blue (430 nm, “OFF”) light. Both FraC(Y138C)–**A** and FraC(W112C)–**A** exhibited hemolytic activity within a short time span upon addition at the tested concentrations and showed a significant difference in the activity of the ON and OFF samples (Figure 3a,b). The analysis of the dose–response curves confirmed that FraC(W112C)–**A** was generally more active (EC50_530nm_(ON) = 0.7 μM, EC50_430nm_(OFF) = 1.1 μM) compared to FraC(Y138C)–**A**, while FraC(Y138C)–**A** (EC50_530nm_(ON) = 2.2 μM, EC50_430nm_(OFF) = 5.2 μM) showed a larger difference in activity of the ON and OFF solutions (Figure 3a,b). For both labeled mutants, the OFF samples, containing predominantly the *trans* isomer of switch **A**, were still relatively active, which is likely a consequence of the presence of the *cis* isomer (the PSD_430nm_ of molecule **7** in Tris buffer is only 76% *trans*, Figure 3e). Besides the possibility that the *trans*-**A** labeled FraC is also somewhat active, the OFF sample contains 24% *cis*-**A** labeled FraC, which might be causing the observed activity. Nevertheless, both mutants exhibited a significant difference in activity of the labeled FraC constructs upon irradiation with two different wavelengths of visible light. Furthermore, the steep dose–response curves (Hill coefficient *n*(FraC112C–*trans*-**A**) = 5.3 and *n*(FraC112C–*cis*-**A**) = 6.5, *n*(FraC138C–*trans*-**A** and −*cis*-**A**) = 3.6), which are inherent to the cooperative nature of the FraC assembly mechanism involving a multimeric pore, are working in favor for this photopharmacological system as a small difference in concentration results in a large response difference. In addition, to confirm the reversibility upon irradiation, a sample of FraC(Y138C)–**A** was subjected to subsequent irradiation cycles with green and blue light, while an aliquot was taken after each irradiation step and its activity was tested on blood (Figure 3c). Consecutive activation of the construct upon λ = 530 nm light irradiation and deactivation upon application of λ = 430 nm light was observed. However, some loss of activity was observed for each of the performed cycles, suggesting slow degradation of the construct upon successive photoswitching cycles.

Finally, we evaluated the cytolytic activity of the visible-light responsive FraC proteins toward human cancer cells. Since both the wild-type FraC and the Y138C mutant exhibited the highest cytolytic activity toward FaDu (hypopharyngeal squamous carcinoma) cells, this cell line was selected for evaluating the cytolytic activity of the visible light-responsive FraC(Y138C)–**A**. As in the hemolytic assays, the samples were preirradiated with the corresponding light (530 nm ON, and 430 nm OFF). At higher concentrations, the activated ON sample of FraC(Y138C)–**A** exhibited higher cytolytic activity compared to the OFF sample (Figure 3d). This result serves as a proof of principle illustrating that the modified FraC nanopore labeled with a water-soluble photoswitch can selectively destroy cancer cells upon activation with visible light.

## Conclusion

PFTs hold great potential for future therapeutic applications, especially due to their high potency in destroying cancer cells. However, the main limitation is their nondiscriminative cytotoxic activity toward normal mammalian cells, which would result in severe off-target side effects of the treatment. To develop PFT-based therapeutics, it is crucial to enable the local activation of their lytic activity with an external, biocompatible trigger, such as visible light.^33^ Here, we aimed to design a fully visible-light responsive PFT by labeling the mutated FraC protein with a water-soluble, green- and blue-light-responsive azobenzene photoswitch in strategically selected locations for manipulating nanopore assembly. The selected azobenzene photoswitch features tetra-*ortho*-fluoro groups which result in favorable photophysical properties and stability, as well as an electron-deficient nature thus enabling functionalization of the molecule via S_N_Ar reaction. This unique synthetic strategy allowed for the later stage introduction of the water-solubilizing sulfonate group. While the water-soluble molecules still proved to require challenging reversed phase purification, the final photoswitch **A** was successfully obtained in sufficient amounts. Both switch **A** and its model analogue **7** exhibited favorable photophysical properties also under aqueous conditions. Molecule **A** was covalently attached to the chosen cysteine mutants of the FraC monomers. The fully visible-light-responsive FraC construct demonstrated a difference in hemolytic activity where the *cis* isomer of **A** (ON sample) was the more active species. Furthermore, FraC(Y138C)–*cis*-**A** was also more active than the blue light-irradiated construct toward FaDu cancer cells, confirming the first report of a fully visible-light-controlled cytolytic protein which destroys cancer cells. Taken together, our strategy paves the way for the development of photocontrolled PFT-based cancer therapeutics.

## Methods

The Supporting Information contains synthetic schemes and procedures, ^1^H, ^13^C and ^19^F NMR spectra, HRMS analysis of compounds, sulfonation reaction optimization, protocol description of protein expression, labeling, purification, and the hemolytic activity assay.

### Safety Statement

No unexpected or unusually high safety hazards were encountered in this study.

### Chemical Synthesis

#### *trans*-4-((4-Bromo-2,6-difluorophenyl)diazenyl)-3,5-difluorobenzenesulfonate (**5**)

Azobenzene **4** (2.0 g, 5.7 mmol) and sodium sulfite (1.0 equiv, 0.72 g, 5.7 mmol) were weighed in a round-bottom flask and were subjected to three vacuum–dry nitrogen cycles. The solids were dissolved in 150 mL of the solvent mixture (water/EtOH, 1:1, v/v), and the solvent was degassed by purging with dry N_2_ for 10 min. The reaction mixture was heated to 50 °C and stirred vigorously overnight. The solvent was partially removed *in vacuo*, and the resulting mixture was extracted with DCM (3 × 100 mL) to remove the remaining starting material which is not soluble in water. The aqueous layer was loaded on Celite and freeze-dried to remove water. The crude product was purified by automatic chromatography on reversed-phase silica gel (C18) with a gradient of the solvent mixture (0–100% 10 mM NH_4_HCO_3_ (aq) in CAN). The obtained fractions were freeze-dried, and the pure product **5** (440 mg, 19%) was isolated as a yellow solid. Due to limited solubility in CAN and formation of aggregates at higher concentrations, the ^13^C spectrum was performed at relatively low concentration. Therefore, we also collected 2D NMR spectra to add to the characterization. Mp > 250 °C. ^1^H NMR (600 MHz, CD_3_CN, drop D_2_O) δ 7.57–7.49 (m, 1H), 7.45 (d, *J* = 8.7 Hz, 1H). ^19^F NMR (565 MHz, CD_3_CN, drop D_2_O) δ −120.44 (d, *J* = 10.6 Hz), −120.49 (d, *J* = 9.9 Hz). ^13^C NMR (151 MHz, CD_3_CN, drop D_2_O) δ: 156.2, 155.7, 154.5, 154.0, 149.4, 131.9, 130.3, 124.7, 117.1, 117.0, 110.8. HR-MS (ESI−) calculated for C_12_H_4_BrF_4_N_2_O_3_S^–^ [M^–^] 410.9057 found: 410.9060. ^1^H NMR (500 MHz, CD_3_CN, drop DCl) δ 7.55 (d, *J* = 8.7 Hz, 1H), 7.47 (d, *J* = 9.0 Hz, 1H). ^19^F NMR (376 MHz CD_3_CN, drop DCl) δ −120.31 (d, *J* = 9.5 Hz), −120.40 (d, *J* = 9.0 Hz). ^13^C NMR (101 MHz, CD_3_CN, drop DCl) δ 157.4 (d, *J* = 5.1 Hz), 156.9 (d, *J* = 3.8 Hz), 154.8 (d, *J* = 5.1 Hz), 154.3 (d, *J* = 3.7 Hz), 148.6, 132.96 (m), 131.0 (t, *J* = 9.4 Hz), 125.7 (t, *J* = 12.4 Hz), 117.9 (d, *J* = 3.9 Hz), 111.5 (dd, *J* = 23.2, 3.3 Hz).

#### *trans*-4-((4-Acetamido-2,6-difluorophenyl)diazinyl)-3,5-difluorobenzenesulfonate (**7**)

Compound **5** (300 mg, 0.726 mmol), acetamide (1.20 eq, 51.5 mg, 0.871 mmol), Cs_2_CO_3_ (3.5 eq, 828 mg, 2.54 mmol), Pd_2_dba_3_ (5 mol %, 33.2 mg, 0.0360 mmol), and XantPhos (20 mol %, 84.0 mg, 0.145 mmol) were weighed into a dry sealable vial equipped with a magnetic stirrer. The solids were subjected to three vacuum–dry nitrogen cycles. Peptide grade DMF (8 mL) was added via a syringe, and the mixture was degassed via five freeze–thaw–pump cycles. (The mixture was frozen by submerging the vial into liquid nitrogen. The frozen solid was put under vacuum, and the valve was closed, leaving a residual vacuum. The reaction mixture was thawed by submerging it into lukewarm water while observing some bubbling. The mixture was again frozen, and the process was repeated.) The reaction mixture was placed under nitrogen and left to stir at 90 °C for 4 h. The crude product mixture was diluted with water/ACN and most of the solvent was removed by rotary evaporation. The remaining solution was transferred with additional water, loaded on Celite, and freeze-dried. The crude product on Celite was dissolved in water and freeze-dried another two times to remove all the remaining traces of DMF. The crude product was purified by automatic chromatography on reversed-phase silica gel (C18) with a gradient of the solvent mixture (0–100% 10 mM NH_4_HCO_3_ (aq) in ACN). The product elutes at 30% ACN. The obtained fractions were freeze-dried, and the pure product was isolated as an orange solid (208 mg, yield = 73%). Mp > 250 °C. ^1^H NMR (600 MHz, CD_3_CN, drop D_2_O) δ 8.94 (s, 1H), 7.46 (d, *J* = 9.7 Hz, 2H), 7.43 (d, *J* = 12.3 Hz, 2H), 2.12 (s, 3H). ^19^F NMR (565 MHz, CD_3_CN, drop D_2_O) δ −119.68 (d, *J* = 12.5 Hz), −122.25 (d, *J* = 9.8 Hz). ^13^C NMR (151 MHz, CD_3_CN, drop D_2_O) δ 170.6, 156.6 (dd, *J* = 7.8, 4.2 Hz), 154.9 (d, *J* = 6.3 Hz), 152.8 (t, *J* = 7.7 Hz), 144.3 (t, *J* = 14.4 Hz), 132.3, 127.7, 111.3 (d, *J* = 3.8 Hz), 111.2 (d, *J* = 3.8 Hz), 103.7 (d, *J* = 3.1 Hz), 103.5 (d, *J* = 3.0 Hz), 24.6. HRMS (ESI−) calcd for C_14_H_8_F_4_N_3_O_4_S^–^: 390.0166, found 390.0179.

#### *trans*-4-((4-Amino-2,6-difluorophenyl)diazenyl)-3,5-difluorobenzenesulfonate (**8**)

Compound **7** (170 mg, 0.43 mmol) was dissolved in concentrated aq. HCl (10 mL, 37%) in an oven-dried sealable vial equipped with a magnetic stirrer. The solution was purged with dry nitrogen for 5 min. The dark purple reaction mixture was heated to 40 °C and left to stir overnight. The reaction mixture was transferred to a flask, and the solvent was removed by rotary evaporation with base (aqueous NaHCO_3_ solution) in the rotary evaporator collection flask. The remaining black solid was dissolved in water and freeze-dried in Corning Falcon conical tubes. The product was used for the next step, as obtained by freeze-drying in a sealable vial equipped with a magnetic stirrer.

#### *trans*-4-((4-(2-Chloroacetamido)-2,6-difluorophenyl)diazenyl)-3,5-difluorobenzenesulfonate (**A**)

Crude aniline **8** (150 mg, 0.4 mmol) was transferred to a sealable vial, and chloroacetic anhydride (2.5 g) was added. The mixture was subjected to three vacuum/dry nitrogen cycles and left under N_2_ to stir at 87 °C for 4 h. The dark purple reaction mixture was dripped into cold ether to precipitate the pure product. The mixture was centrifuged (4000 rpm) and washed multiple times with cold ether. The pure product was obtained as a dark purple solid (200 mg, yield >95%). Mp > 250 °C. ^1^H NMR (600 MHz, DMSO-*d*_6_) δ 11.20 (s, 1H), 7.57 (d, *J* = 12.0 Hz, 2H), 7.40 (d, *J* = 12.0 Hz, 2H), 4.37 (s, 2H). ^19^F NMR (565 MHz, DMSO-*d*_6_) δ −118.06 (d, *J* = 12.6 Hz), −120.82 (d, *J* = 10.1 Hz). ^13^C NMR (151 MHz, DMSO-*d*_6_) δ 166.0, 156.6 (d, *J* = 6.4 Hz), 155.4–154.4 (m), 153.2 (d, *J* = 3.8 Hz), 152.1 (d, *J* = 7.0 Hz), 142.9 (t, *J* = 14.0 Hz), 130.7, 126.4, 110.2 (d, *J* = 3.6 Hz), 110.0 (d, *J* = 3.4 Hz), 103.1 (d, *J* = 3.0 Hz), 103.0–102.9 (m), 43.5. HRMS (ESI−) calcd for C_14_H_7_ClF_4_N_3_O_4_S^–^: 423.9787, found 423.9790.

### Protein Expression, Labeling, and Purification

The FraC(W112C) and FraC(Y138C) constructs were created and expressed as described previously.^50^ In brief, the constructs were transformed into the electrocompetent E. cloni EXPRESS BL21(DE3) cells and cultured in 1 L of 2×YT medium supplemented with 100 μg/mL ampicillin at 37 °C while shaking at 200 rpm, until reaching an OD_600_ of ∼0.8. Protein expression was induced by addition of 0.5 mM isopropyl β-d-thiogalactopyranoside (IPTG) and subsequent culturing overnight at 25 °C while shaking at 200 rpm.

The cells were harvested by centrifugation at 8000*g* for 10 min at 4 °C and subjected to one freeze–thaw cycle at −80 °C to increase susceptibility to cell lysis. Cell pellets, from 50 mL of cell culture, were resuspended in 10 mL of lysis buffer (50 mM Tris-HCl pH 7.5, 150 mM NaCl, 4 M urea, 1 mM MgCl_2_, 2.5 mM TCEP, 10 μg/mL lysozyme, 0.1 U/mL DNase) and incubated for 20 min at RT while rotating at 25 rpm. The cells were further disrupted via probe sonication (Brandson) for 2 min at 25% output power. Cell debris were removed via centrifugation at 8000*g* for 30 min at 4 °C. The supernatant was incubated with 300 μL (bead volume) of Ni-NTA resin (Qiagen) for 10 min while rotating at 10 rpm. The incubated Ni-NTA resin was loaded onto a gravity flow column (Bio-Rad) and washed with 15 mL of wash buffer 1 (50 mM Tris-HCl pH 7.5, 150 mM NaCl, 10 mM imidazole, and 2.5 mM TCEP) and 20 mL of nitrogen degassed wash buffer 2 (50 mM NaH_2_PO_4_ pH 9.5, 150 mM NaCl). Wash buffer 2 is used to remove Tris-HCl, imidazole, and TCEP that can inhibit the labeling of the light switch. The FraC constructs were eluted from the Ni-NTA resin with 3 times 250 μL of nitrogen degassed elution buffer (50 mM NaH_2_PO_4_ pH 9.5, 150 mM NaCl, 200 mM EDTA) in a darkroom in light protective microtubes. After each elution step, the protein concentration was immediately determined spectroscopically (ε_280nm_(FraC)= 43.9 × 10^3^ M^–1^ cm^–1^) and the azobenzene **A** was added in a 1:50 ratio (protein/azobenzene). The stock solution of switch **A** was previously freshly prepared in 1/1 solution of water/DMSO. The labeling mixture was incubated with the protein for 2 h while shaking and preventing any ambient light exposure (Figure S1).

The excess photoswitch was separated from the labeled protein with two desalting columns (HiTrap Desalting column, Cytiva) connected to an Äkta pure chromatography system. The sample was applied to the HiTrap desalting columns equilibrated with running buffer (15 mM Tris-HCl pH 9.5). Protein elution was monitored by measuring absorbance at 280, 365, and 420 nm wavelengths. The first eluted peak corresponds to the FraC monomers labeled with the visible light switch (Figure S2). Collected elution fractions of the first peak were combined and concentrated by using the rotary evaporator at 35–40 °C. Protein concentration was determined by UV-spectroscopy while approximating the same extinction coefficient as unlabeled FraC (ε_280nm_(FraC)= 43.9 × 10^3^ M^–1^ cm^–1^). Labeled FraC monomers were stored at 4 °C until further use.

### Cancer Cell Cytolytic Activity Assay

#### Cancer Cells

The human hypopharyngeal squamous carcinoma cell line FaDu was obtained from the American Type Culture Collection (HTB-43, ATCC). The adherently growing FaDu cancer cells were cultured in DMEM medium (Lonza) supplemented with 10% fetal calf serum (FCS, ThermoFisher) at 37 °C in a humidified 5% CO_2_ atmosphere.

#### Assessment of Anticancer Activity of FraC Variants

The capacity of the various FraC constructs to eliminate FaDu cancer cells was assessed by flow cytometry using the annexin-V-FITC/propidium iodide staining method. In short, FaDu cancer cells were cultured in 48-well culture plates (1.5 × 10^4^ cells/well) and treated (or not) with the indicated concentrations of control buffer, FraC-138C-ON, or FraC-138C-OFF for 2 h at 37 °C. Subsequently, cancer cells were harvested from the respective wells and evaluated for the percentage cells Annexin-V/PI positive by flow cytometry.

## Supporting Information Available

Synthetic schemes and procedures, ^1^H, ^13^C, and ^19^F NMR spectra, HRMS analysis of compounds, sulfonation reaction optimization, protocol description of protein expression, labeling, purification, and the hemolytic activity assay (PDF)
