# Supplementary material for: Visible Light Control over the Cytolytic Activity of a Toxic Pore-Forming Protein
Source: ACS Chem Biol. 2024 Feb 6;19(2):451–61. doi: 10.1021/acschembio.3c00640 (PMC10877574; doi:10.1021/acschembio.3c00640)
Supplement: Supplementary file 1 — cb3c00640_si_001.pdf [file cb3c00640_si_001.pdf]

Supporting information for

Visible Light Control over the Cytolytic Activity of a Toxic Pore-forming Protein

Jana Volarić,[a] Nieck J. van der Heide,[b] Natalie L. Mutter,[b,†] Douwe F. Samplonius,[c] Wijnand Helfrich,[c] Giovanni Maglia,[b,]* Wiktor Szymanski,[a,d,]* Ben L. Feringa[a,]*

e-mail : giovanni.maglia@rug.nl, w.c.szymanski@rug.nl, b.l.feringa@rug.nl

[a] Stratingh Institute for Organic Chemistry, University of Groningen, 9747 AG Groningen, The Netherlands

[b] Groningen Biomolecular Sciences and Biotechnology Institute, University of Groningen, Groningen 9747 AG, The Netherlands

[c] Department of Surgery, Translational Surgical Oncology, University of Groningen, University Medical Center Groningen, Hanzeplein 1, 9713 GZ, Groningen, The Netherlands

[d] Department of Radiology, Medical Imaging, Center, University of Groningen, University Medical Center Groningen, 9713 GZ Groningen, The Netherlands

1 Experimental Section

1.1 General information

All chemicals for synthesis were obtained from commercial sources and used as received unless stated otherwise. Technical grade solvents were used for extraction and chromatography. Thin Layer Chromatography (TLC) was performed using commercial Kiesegel 60 F254 silica gel plates with fluorescence-indicator UV254 (Merck, TLC silica gel 60 F254). For detection of components, UV light at λ=254 nm or λ=365 nm was used. Alternatively, oxidative staining was performed using a basic solution of potassium permanganate in water or aqueous cerium phosphomolybdic acid solution (Seebach's stain). Merck silica gel 60 (230–400 mesh ASTM) was used in normal phase flash chromatography. Büchi Reveleris® X2 automatic column was used with Büchi EcoFlex silica columns (4 - 40 g, 40−63 µM, 60 Å).
Spectroscopic measurements were made in Uvasol® grade solvents using a quartz cuvette (path length 10.0 mm). UV-Vis measurements were performed on an Agilent 8453 UV-Visible absorption Spectrophotometer. UV-Vis irradiation experiments were carried out using a custom-built (Prizmatix/Mountain Photonics) multi-wavelength fiber coupled LED-system (FC6-LED-WL) with LED lights (425A and 530B), 530 nm and 430 nm LED light source (3x 530 nm, 3x 430 nm, LED Nichia NCSB219B-V1, Sahlmann Photochemical Solutions). The temperature was controlled with a Quantum Northwest TC1 temperature controller. The data was processed using Agilent UV-Vis ChemStation B.02.01 SP1, Spectragryph 1.2, OriginPro 2016 and all images were assembled in Adobe Illustrator.
NMR spectra were obtained using Agilent Technologies 400-MR (400/54 Premium Shielded) (400 MHz) and Bruker Innova (1H: 600 MHz, 13C: 151 MHz) spectrometers at room temperature (22-24 °C). Chemical shift values (δ) are reported in parts per million (ppm) with the solvent resonance as the internal standard ($CDCl_3$: δ 7.26 for ^{1}H, δ 77.16 for ^{13}C; DMSO: δ 2.05 for ^{1}H, δ 39.52 for ^{13}C, CD_3OD: δ 3.31 for ^{1}H, δ 49.0 for ^{13}C). The following abbreviations are used to indicate signal multiplicity: s (singlet), d (doublet), t (triplet), q (quartet), m (multiplet), brs (broad signal) or dd (doublet of doublets).
Exact mass spectra were recorded on an LTQ Orbitrap XL (ESI+). All reactions requiring an inert atmosphere were carried out under a nitrogen atmosphere using oven dried glassware and standard Schlenk techniques. Dichloromethane and toluene were used from solvent purification system using an MBraun SPS-800 column. Melting points were determined using Stuart analogue capillary melting point SMP11 apparatus. All errors are given as standard deviations.
NB. For all sulfonated compounds, purification proved to be challenging and tedious, requiring reversed phase chromatography using mildly basic buffer/ACN mixtures as eluents. The buffer used, ammonium hydrogencarbonate, was chosen for easy removal of the salt via freeze-drying. Furthermore, decomposition of the photoswitches was observed upon removal of water on the rotary evaporator at higher temperatures, thus requiring complete removal of the solvent exclusively by a freeze-drying procedure.

1.2 Chemical Synthesis

5-bromo-1,3-difluoro-2-nitrosobenzene (2)[1]

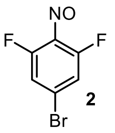

2

Aniline **1** (7.0 g, 34 mmol) was dissolved in 70 mL of chloroform, and in a separate flask Oxone© (3.0 eq, 31 g, 100 mmol) was dissolved in 90 mL of water. The solutions were combined in a 500 mL round-bottom flask and the resulting biphasic mixture was stirred vigorously for 24 h at room temperature. The biphasic reaction mixture was extracted with chloroform (3 x 100 mL), the combined organic phases were washed with 1 M aq. HCl (2 x 100 mL), aq. $NaHCO_3$ (2 x 100 mL), water (130 mL) and brine (2 x 90 mL), The organic layer was dried over $MgSO_4$ and the solvent was removed *in vacuo*. The crude product was obtained as a light green solid (6.8 g, yield = 90%) and used as obtained for the next step. The spectroscopic data is in accordance with the literature.[1]

Mp. 87–90 °C; **FTIR (ATR):** 1601 cm^{-1} (s, C=C), 1431 cm^{-1} (s, N-O), 1275 cm^{-1} (s, C-N), 1060 cm^{-1} (s, C-N), 542 cm^{-1} (m, C-F); **^{1}H NMR** (400 MHz, $CDCl_3$) δ 7.38–7.31 (m, 2H), ^{13}C-NMR (101 MHz, $CDCl_3$) δ 153.5 (dd, J = 274.2 Hz), 130.7 (t), 117.1 (dd, J = 23.1 Hz), ^{19}F-NMR (376 MHz, $CDCl_3$*d*) δ -128.66 (d, *J* = 8.2 Hz).

***trans*-1-(4-bromo-2,6-difluorophenyl)-2-(2,4,6-trifluorophenyl)diazene (4)**

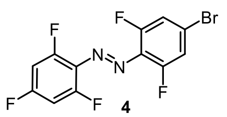

Nitroso compound **2** (6.0 g, 27 mmol) was dissolved in 100 mL of the solvent mixture (toluene/AcOH/TFA, 40:40:6, v/v) and 2,4,6-trifluoroaniline **3** (1.0 eq, 3.9 g, 27 mmol) was added. The reaction mixture was then stirred at room temperature for 24 h. The solvent was removed by rotary evaporation from the red-brown solution, with base (aqueous $NaHCO_3$ solution) in the rotary evaporator collection flask. The crude product mixture was purified via column chromatography on silica with pentane as the eluent. The product was isolated as a red solid (7.9 g, yield=83%).

Mp. 94-96 °C. **HR-MS (ESI+)** calculated for $C_{12}H_5BrF_5N_2^+$: 350.9551 found: 350.9554.

^{1}H NMR (400 MHz, $CDCl_3$) δ 7.28-7.23 (m, 2H), 6.88-6.80 (t, J = 8.8 Hz, 2H).**^{19}F NMR (376 MHz, $CDCl_3$)** δ -102.04 (p, J = 8.5 Hz), -115.95 (t, J = 8.8 Hz), -118.98 (d, J = 8.6 Hz). **^{13}C NMR (101 MHz, $CDCl_3$)** δ 165.1 (t, J = 15.1 Hz), 162.6 (td, J = 15.2, 14.9, 13.7 Hz), 158.5 – 158.1 (m), 157.2 (d, J = 5.1 Hz), 155.7 (dd, J = 15.2, 6.2 Hz), 154.6 (d, J = 5.9 Hz), 131.2, 124.5 (t, J = 11.9 Hz), 117.0 (d, J = 27.4 Hz), 102.4 – 101.7 (m).

^{1}H NMR (400 MHz, DMSO-*d*$_6$) δ 7.77 (d, J = 9.6 Hz, 1H), 7.51 (t, J = 9.7 Hz, 1H); **^{19}F NMR (376 MHz, DMSO-*d*$_6$)** δ -101.40 (p, J = 9.1 Hz), -116.88 (t, J = 9.7 Hz), -119.75 (d, J = 9.7 Hz). **^{13}C NMR (101 MHz, DMSO-*d*$_6$)** δ 164.5 (t, J = 15.7 Hz), 161.9 (t, J = 15.9 Hz), 157.1 (dd, J = 16.0, 6.5 Hz), 156.0 (d, J = 5.1 Hz), 154.5 (dd, J = 16.1, 6.5 Hz), 153.4 (d, J = 5.0 Hz), 129.8 (t, J = 9.7 Hz), 124.4 (t, J = 12.4 Hz), 117.1 (d, J = 23.7 Hz), 102.7 – 102.0 (m).

1.3 Sulfonation reaction conditions

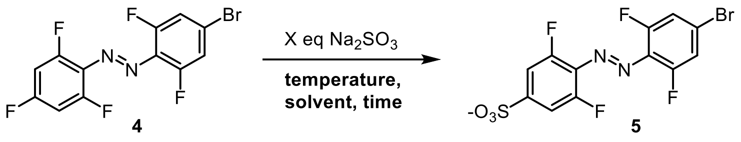

We conducted several qualitative test reactions where we looked for formation of water-soluble products during the S_NAr reaction of non-water soluble azobenzene **4** with sodium sulfite at different reaction conditions. If the sulfonation occurs, sulfonated azobenzene eluted on reversed phase silica. However, since over-sulfonation can take place and we have previously observed formation of complex mixtures of water-soluble products, in these experiments we specifically aimed to optimize the reaction conditions to yield the least possible number of water-soluble side products. Therefore, here we qualitatively investigated the number of water-soluble products formed by reversed phase TLC and in some examples determined the conversion. To summarize the results, the use of solvent mixtures containing 40-60% of water and a polar solvent, such as ethanol, ACN or DMF, resulted in less complex water-soluble product mixtures with the highest content of the mono sulfonated product. This is likely due to the optimal solvent mixture required to dissolve both the non-polar, aromatic azobenzene and the highly polar water-soluble sodium sulfite salt. While higher temperatures increased the conversion of the sulfonation reaction, unfortunately they resulted in complex mixtures of multi-sulfonated products and by-products which were extremely difficult to separate. Therefore we opted for conditions yielding the highest amount of the mono-sulfonated compound **5**, namely 1/1 = water/ACN at 50 °C (overnight), resulting in minimal formation of multi-sulfonated species, especially since the starting material could easily be recovered by extraction of the crude mixture with DCM.

1.4 Protein expression, labelling and purification

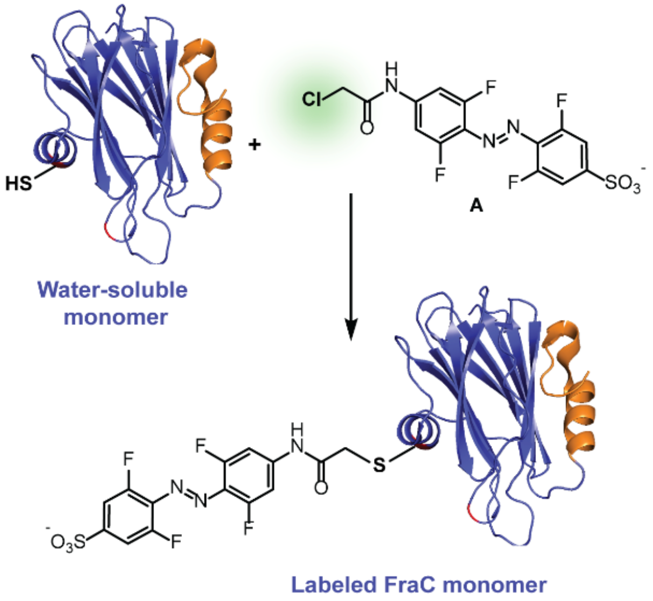

Figure S1. Scheme representing the attachment reaction of visible light-responsive switch **A** to the thiol group of cysteine in the water-soluble conformation of the FraC monomer. The alpha helix which gets extended into the lipid bilayer is highlighted in orange (PDB 4TSP[2]).

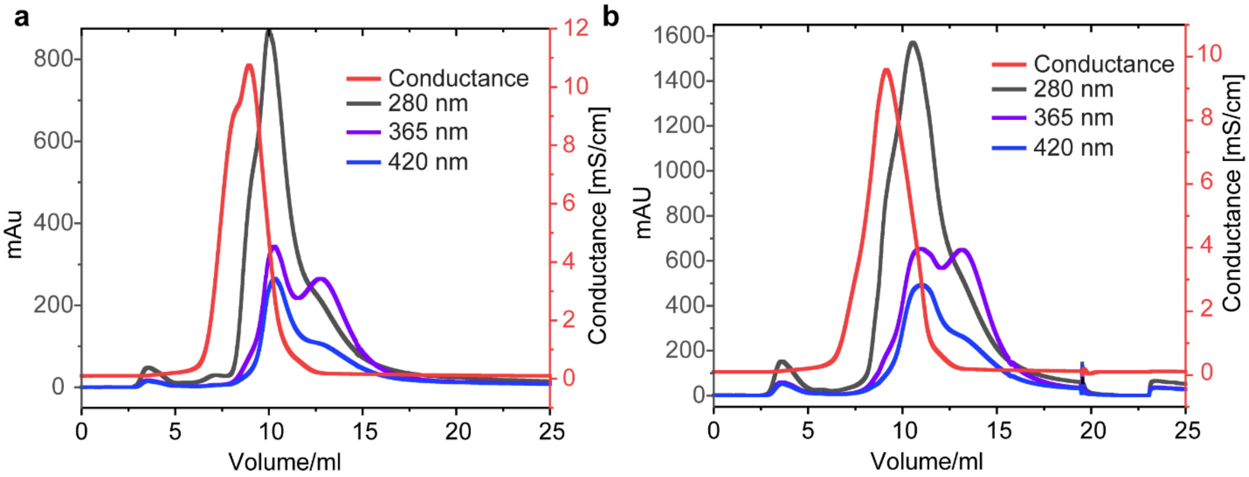

Figure S2. Chromatogram obtained from eluting FraC mutants labelled with azobenzene **A** via a HiTrap desalting column (Cytiva) equilibrated with running buffer (15 mM Tris-HCl pH: 9.5) on the Äkta pure chromatography system while measuring absorbance at 280 nm, 365 nm and 420 nm: **a** FraC-Y138-**A**, **b** FraC-W112-**A**.

1.5 Haemolytic activity assay

Defibrinated sheep blood (Fisher Scientific) was washed several times with wash buffer (15 mM Tris-HCl pH 7.5, 150 mM NaCl) by centrifugation (8000 rpm for 10 s) until the supernatant was clear. The washed blood was resuspended and diluted in wash buffer to an OD_{650} of 0.8-0.9. The activity assays were performed in a dark room under red light. The diluted blood (100 µL) was added to a 96-well plate containing several solutions with different concentrations of toxin (labelled FraC monomers) in either *cis* or *trans*-state. Immediately after adding the red blood cells suspension, the haemolytic activity was measured by monitoring the decrease in OD_{650} using the Synergy H1 Hybrid-Multimode reader (BioTek). Percentage of haemolysis was calculated as followed for a specific time point:

$$\% \text{ hemolysis} = 100*(Abs_{C\text{-}FraC} - Abs_{buffer})/(Abs_{triton} - Abs_{buffer}).$$

where $Abs_{C\text{-}FraC}$ is the absorbance measured for the blood sample were the labelled FraC monomers were added, Abs_{buffer} is the absorbance measured for the blood sample were only buffer was added (negative control) and Abs_{triton} is the absorbance measured for the blood sample were Triton, a strong detergent which causes complete lysis of blood cells, was added (positive control). Photoisomerization of the visible light switch to the *cis*-state was achieved by irradiation for 30 min with the 530 nm wavelength LED and to the *trans*-state by irradiation for 30 min with the 430 nm wavelength LED.

For the reversibility test, the same sample of labelled FraC monomers was consecutively irradiated with 430 nm and 530 nm lamps for 30 min, while removing three aliquots after each irradiation step. The aliquoted samples were measured in the haemolytic activity assay.

2 References

(1) Yang, X.; Ma, G.; Zheng, S.; Qin, X.; Li, X.; Du, L.; Wang, Y.; Zhou, Y.; Li, M.; Li, M. Optical Control of CRAC Channels Using Photoswitchable Azopyrazoles. *J. Am. Chem. Soc.* **2020**, *142* (20), 9460–9470.

(2) Tanaka, K.; Caaveiro, J. M. M.; Morante, K.; González-Mañas, J. M.; Tsumoto, K.; González-Manãs, J. M.; Tsumoto, K. Structural Basis for Self-Assembly of a Cytolytic Pore Lined by Protein and Lipid. *Nat. Commun.* **2015**, *6* (1), 6337.

3 Appendix

3.1 Analysis data of new compounds

***trans*-1-(4-bromo-2,6-difluorophenyl)-2-(2,4,6-trifluorophenyl)diazene (4)**

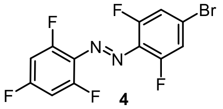

4

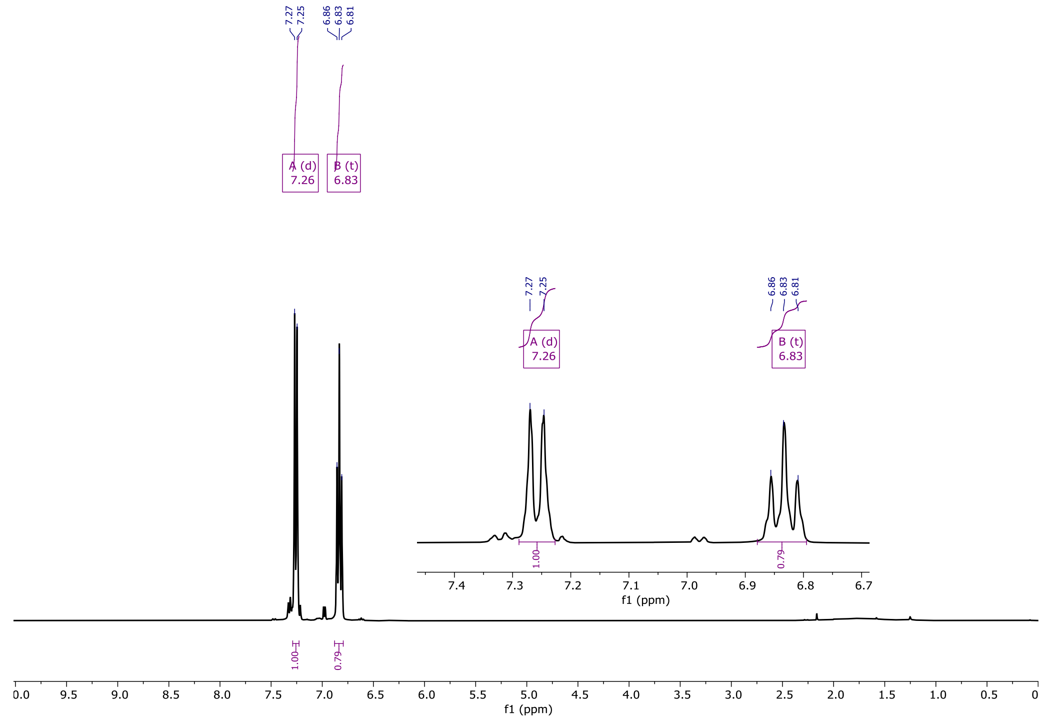

Figure S3. ^{1}H-NMR of compound **4** in $CDCl_3$.

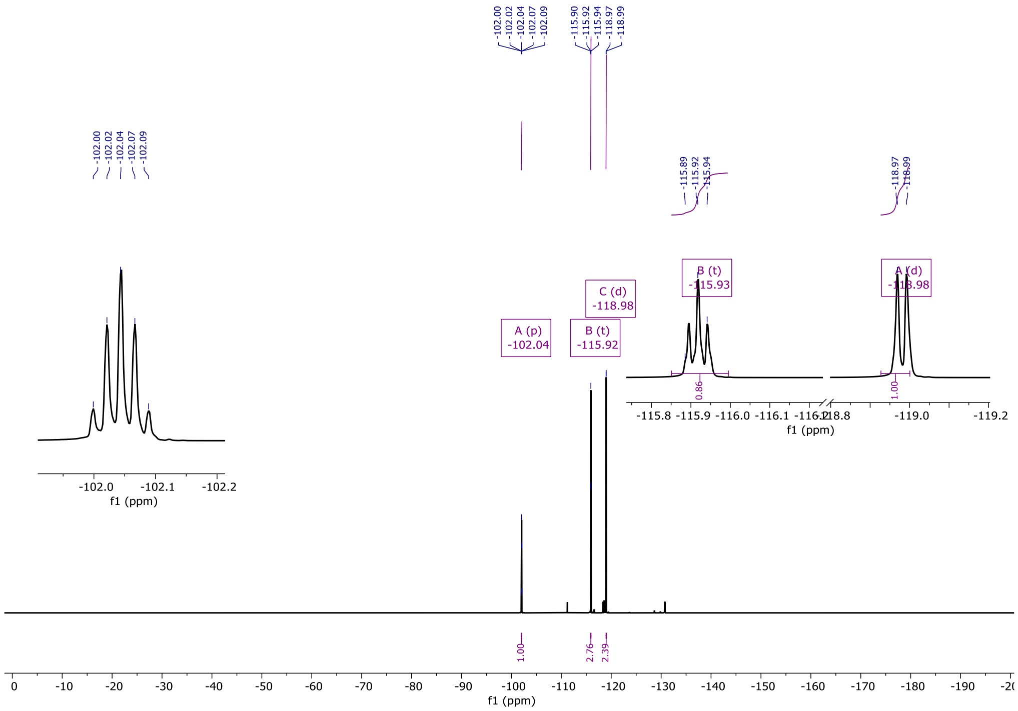

Figure S4. ^{19}F-NMR of compound **4** in $CDCl_3$.

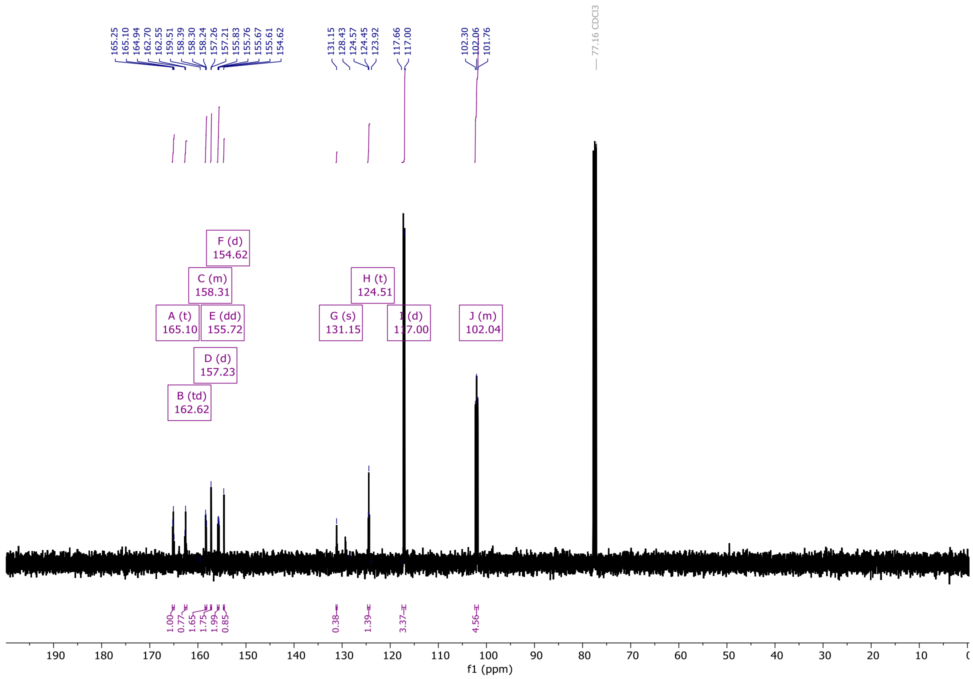

Figure S5. ^{13}C-NMR of compound **4** in $CDCl_3$.

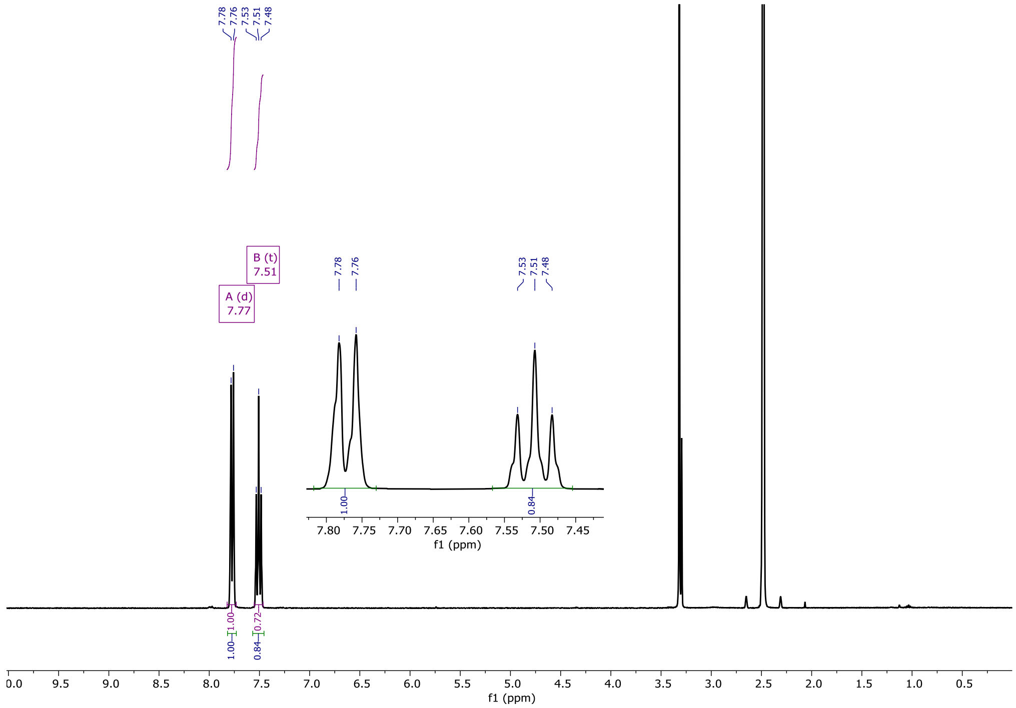

Figure S6. ^{1}H-NMR of compound **4** in DMSO-d_6.

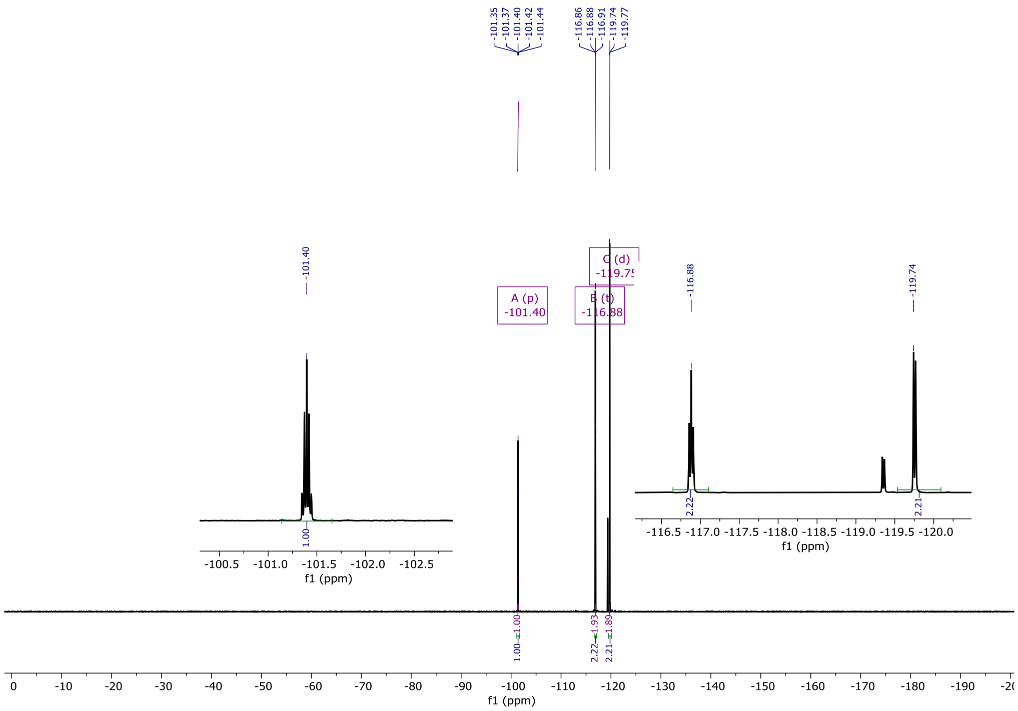

Figure S7. ^{19}F-NMR of compound **4** in DMSO-d_6.

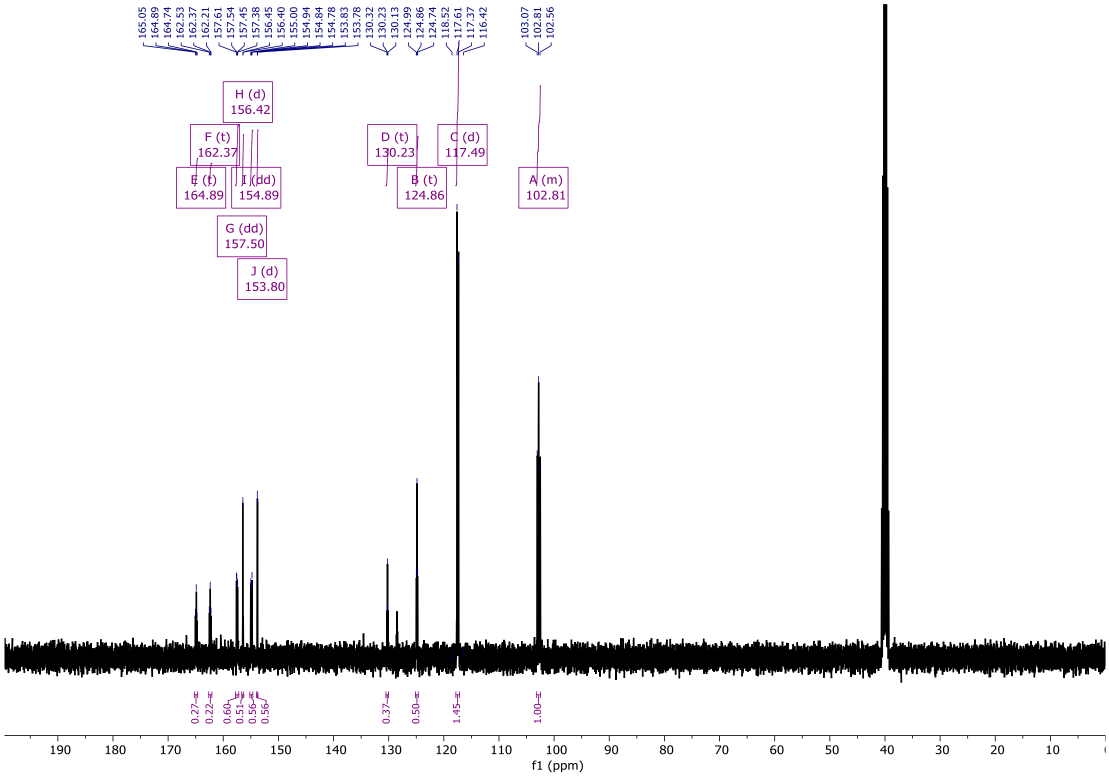

Figure S8. ^{13}C-NMR of compound **4** in DMSO-d_6.

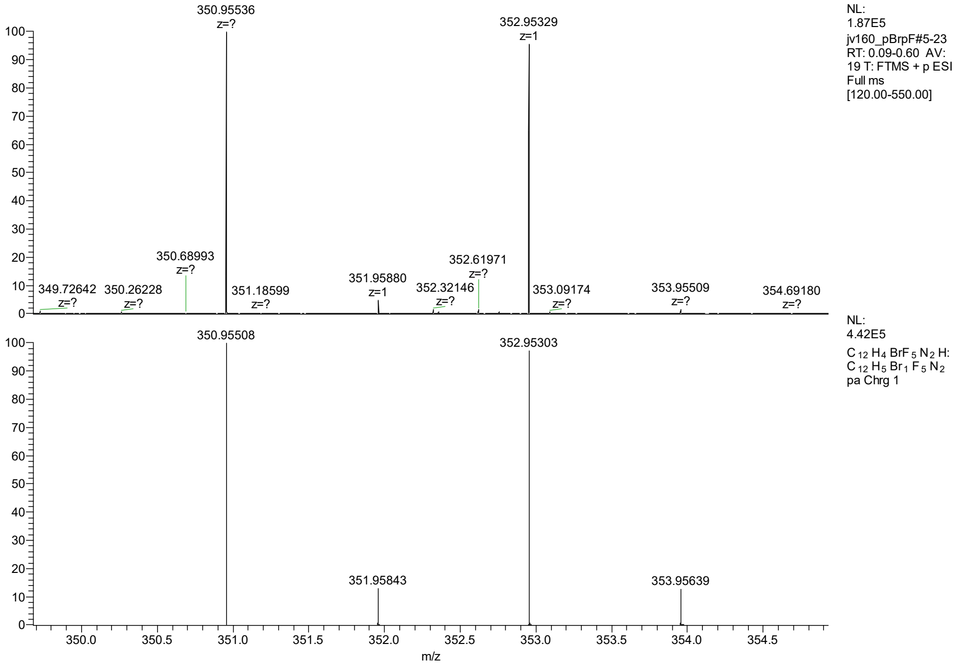

Figure S9. HRMS analysis of compound.

***trans*-4-((4-bromo-2,6-difluorophenyl)diazenyl)-3,5-difluorobenzenesulfonate (5)**

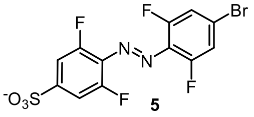

* Due to limited solubility in ACN and formation of aggregates at higher concentrations, the ^{13}C spectrum is performed at relatively low concentration. Therefore we also collected 2D NMR spectra to add to the characterization.

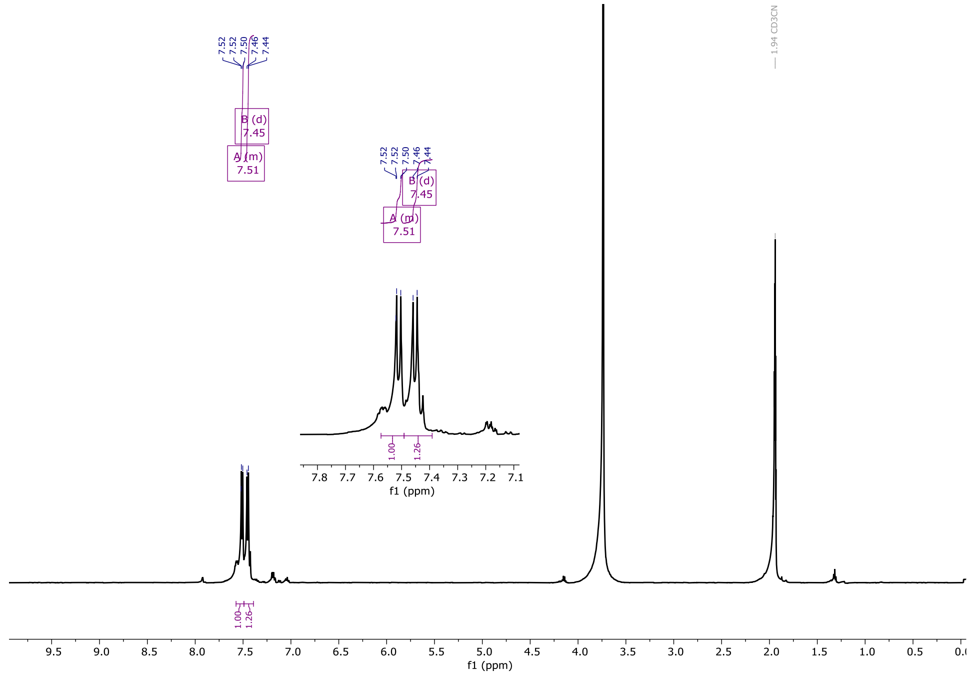

Figure S10. ^{1}H-NMR of compound **5** in CD_3CN, drop D_2O.

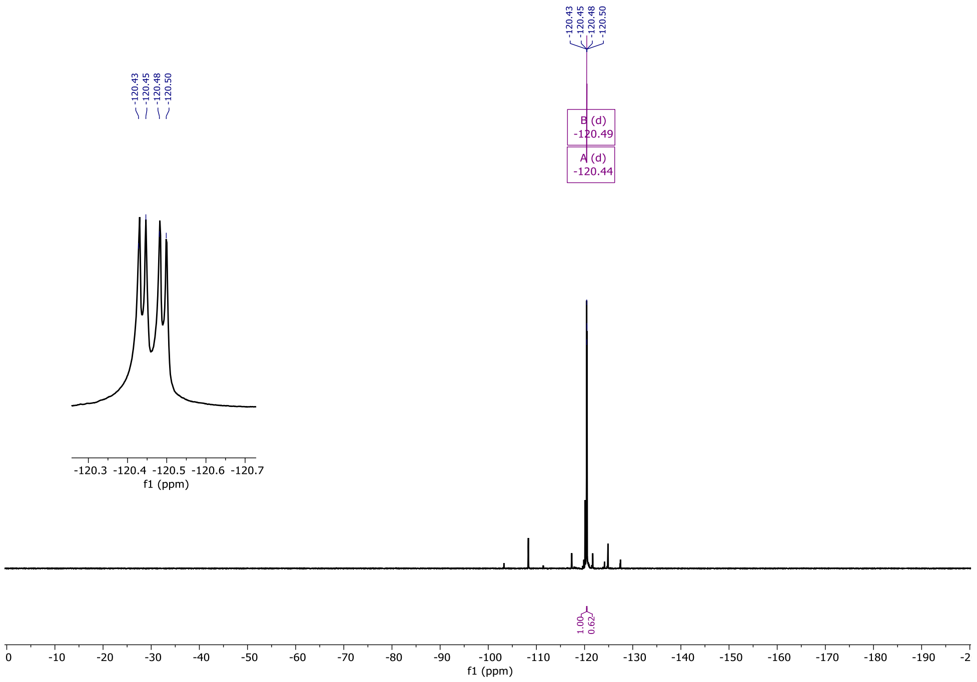

Figure S11. ^{19}F-NMR of compound **5** in CD_3CN, drop D_2O.

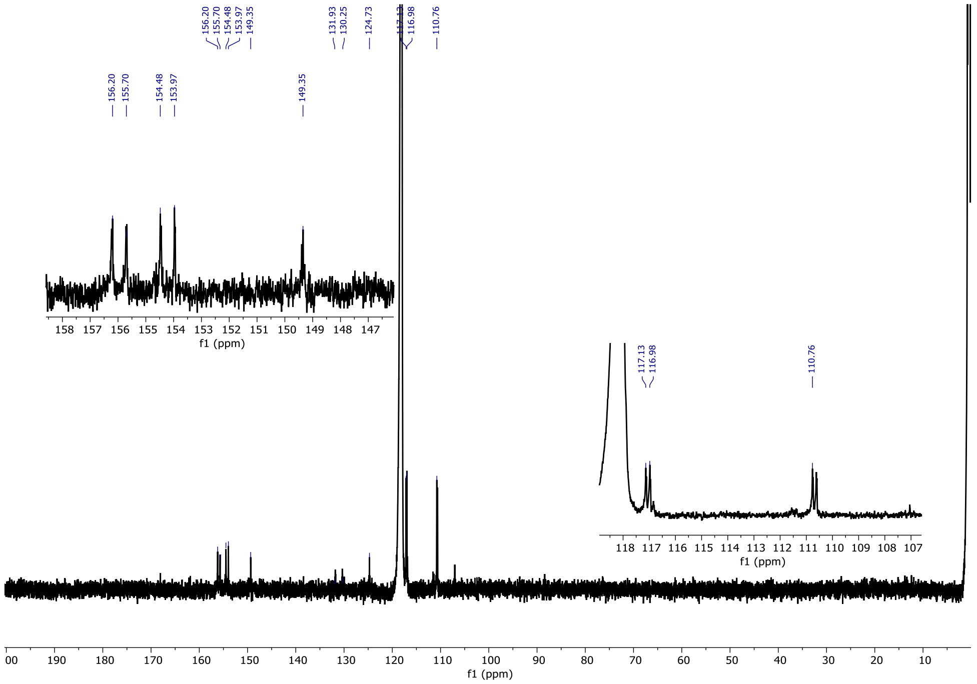

Figure S12. ^{13}C-NMR of compound **5** in CD_3CN, drop D_2O.

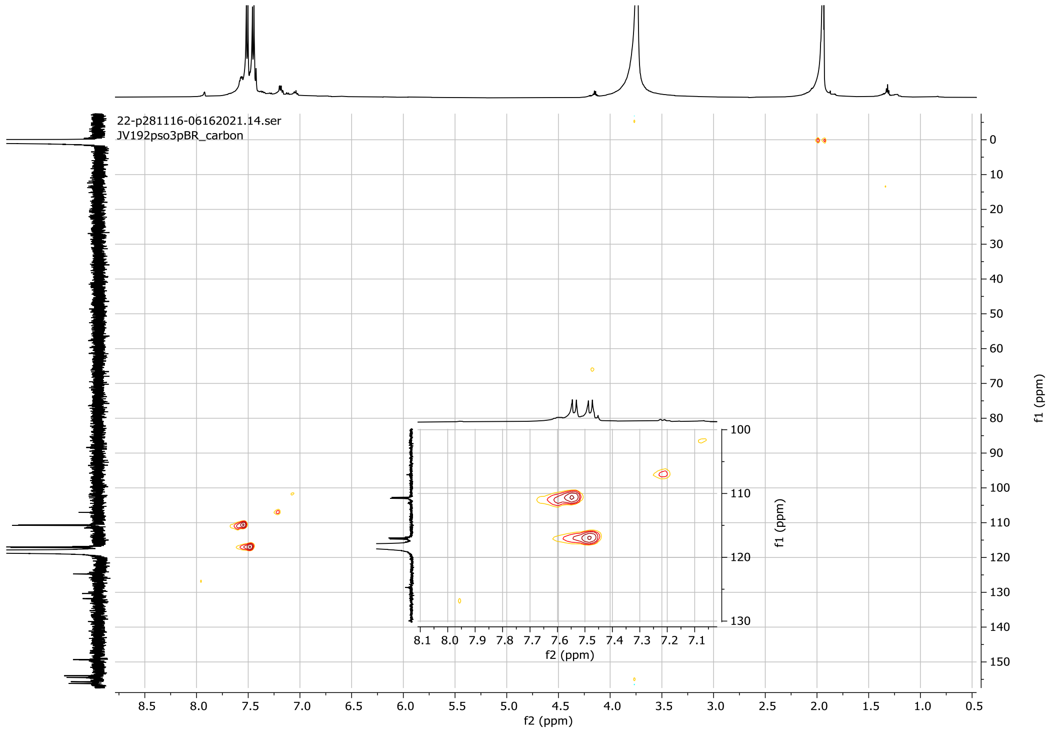

Figure S13. HSQC 2D-NMR of compound **5** in CD_3CN, drop D_2O.

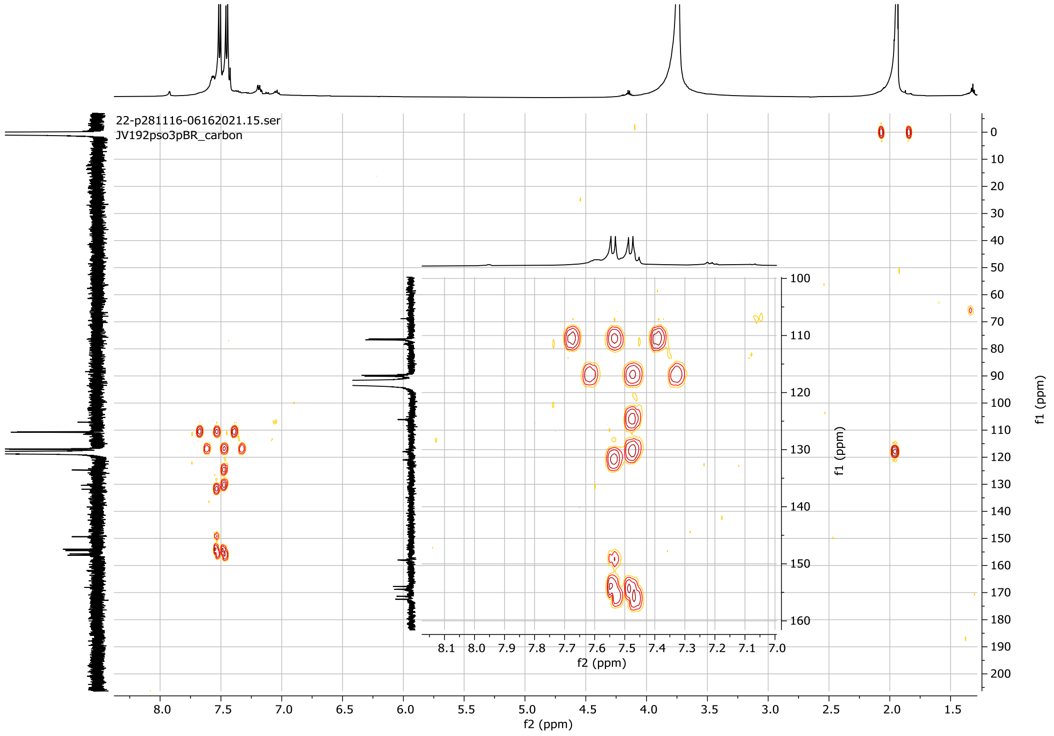

Figure S14. HMBC 2D-NMR of compound **5** in CD_3CN, drop D_2O.

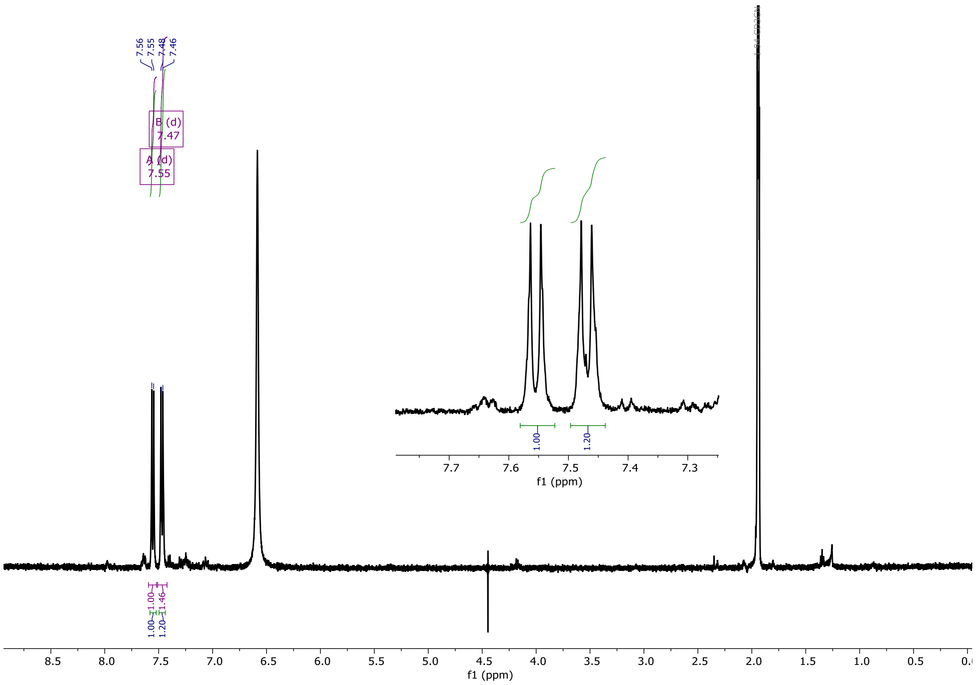

Figure S15. ^{1}H-NMR of compound **5** (with impurities present) in CD_3CN with a drop DCl.

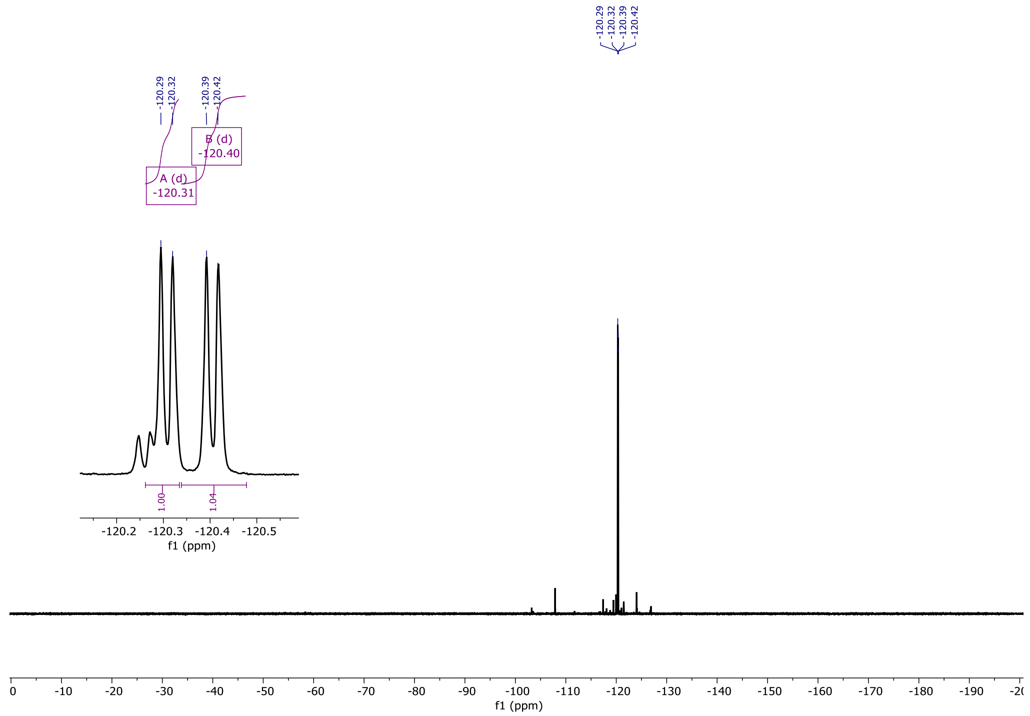

Figure S16. ^{19}F-NMR of compound **5** (with impurities present) in CD_3CN with a drop DCl.

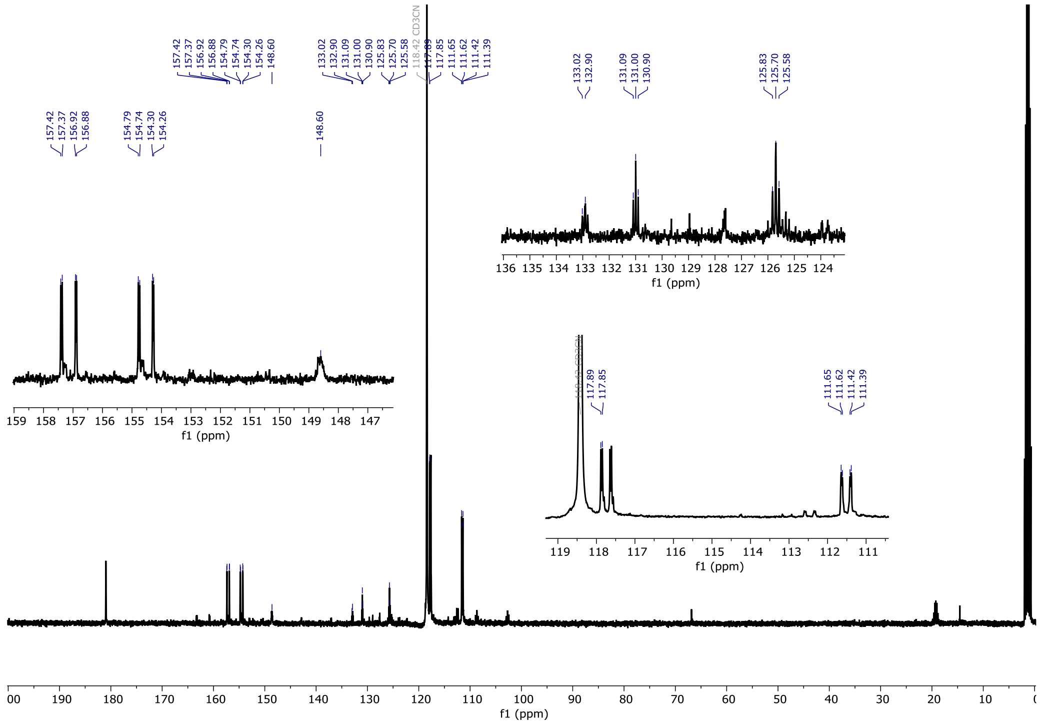

Figure S17. ^{13}C-NMR of compound **5** (with impurities present, peak at 180 ppm) in CD_3CN with a drop DCl.

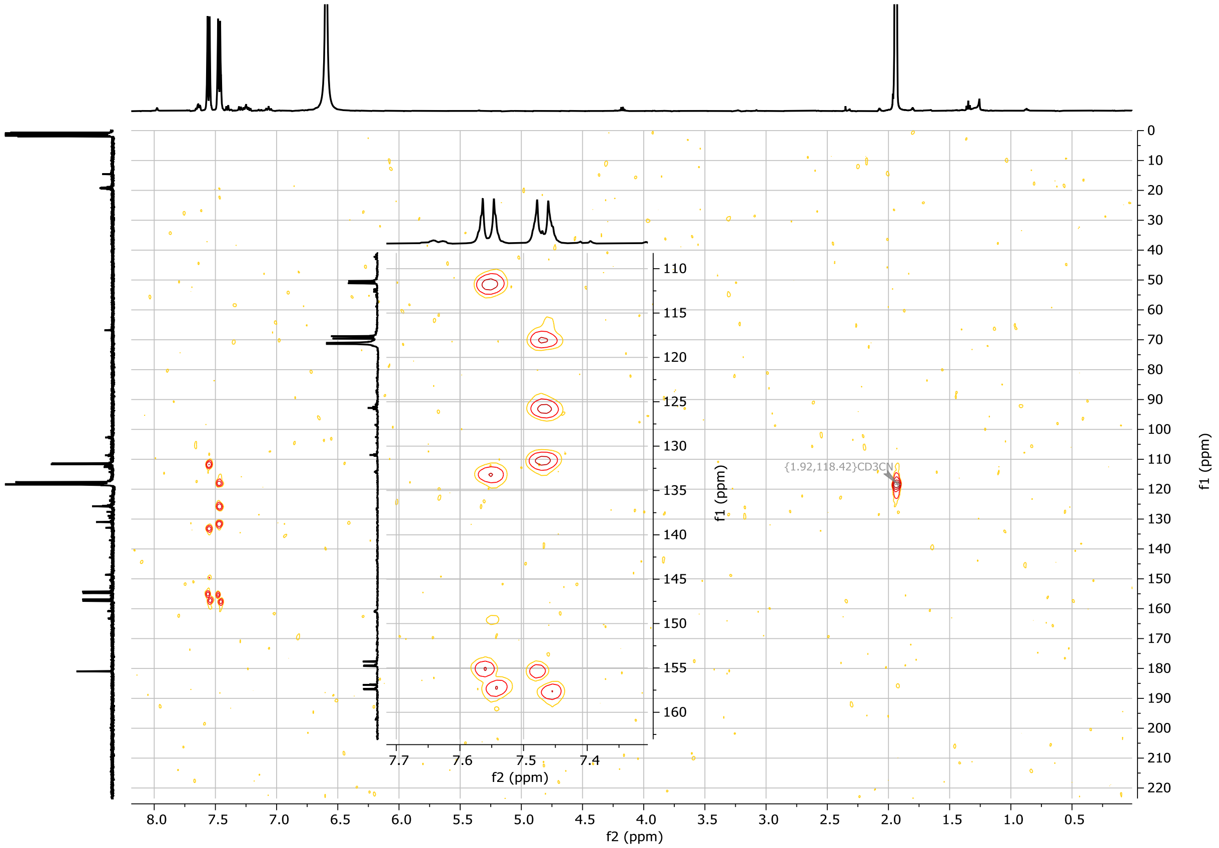

Figure S18. HMBC 2D-NMR of compound **5** (with impurities present) in CD_3CN with a drop DCl.

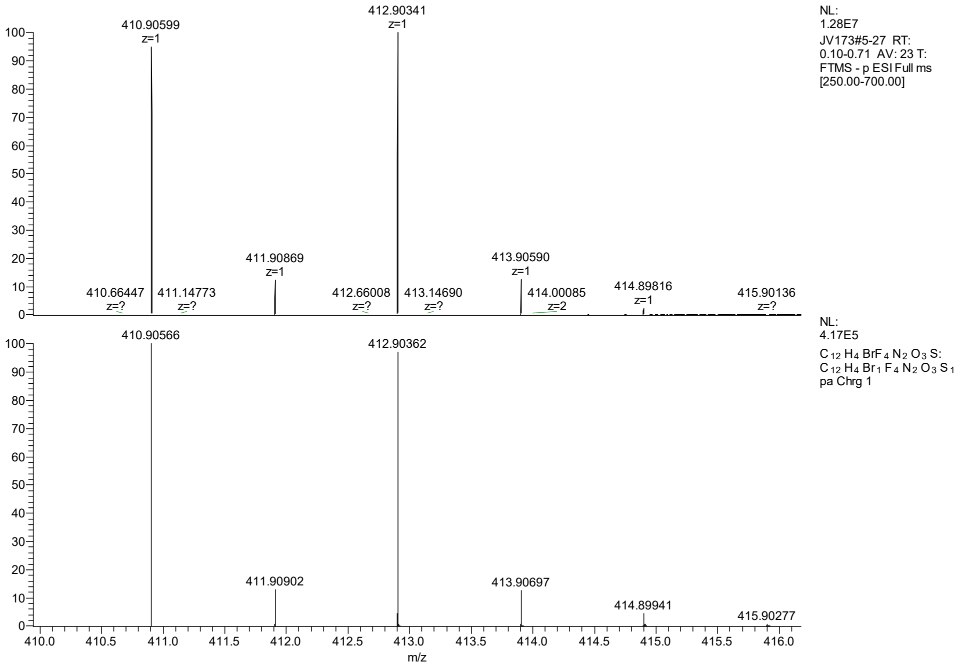

Figure S19. HRMS analysis of compound **5**.

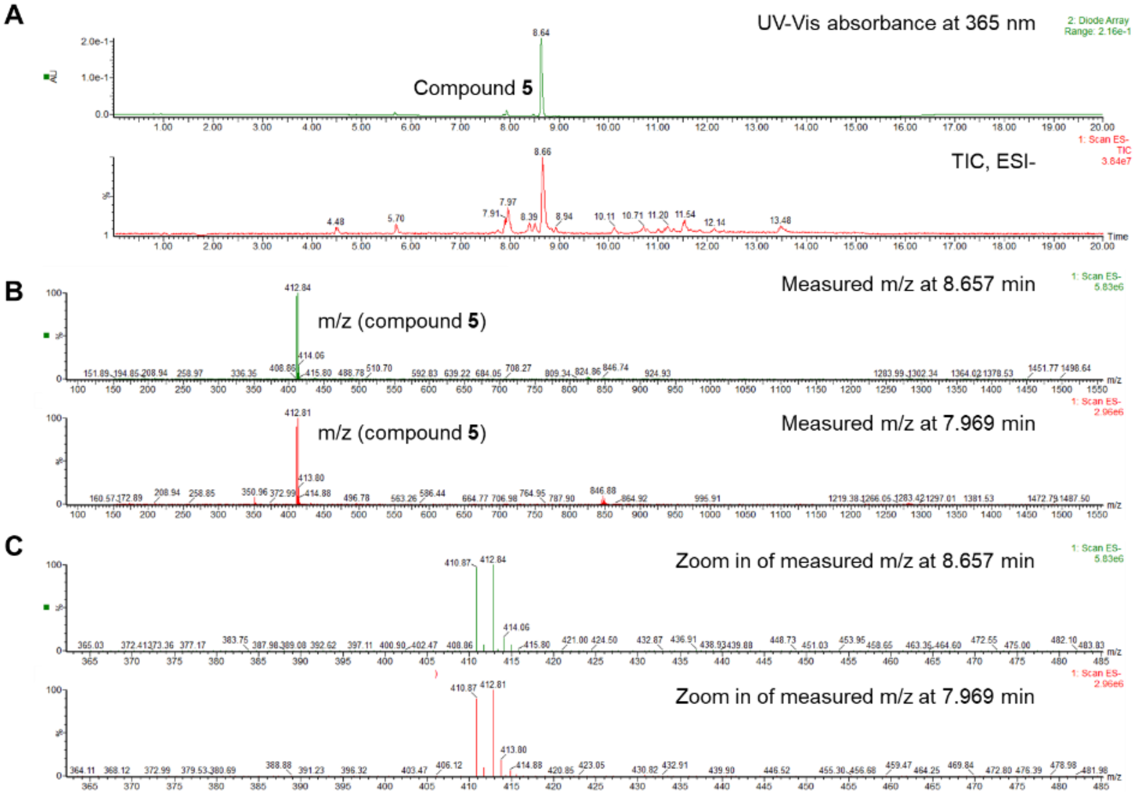

Figure S20. LCMS traces of isolated compound **5** measured in negative mode (Conditions: 0.1% NH_3 in water and 0.1% NH_3 in ACN as eluents, HSST3 C18 column). **A)** Measured absorbance at 365 nm and the total ion current (TIC) measured in negative mode. **B)** Measured m/z of observed peaks at noted retention times and the zoom in of the observed m/z (**C**) to visualize the isotope pattern due to the presence of bromine.

***trans*-4-((4-acetamido-2,6-difluorophenyl)diazenyl)-3,5-difluorobenzenesulfonate (7)**

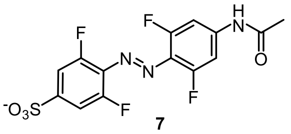
7

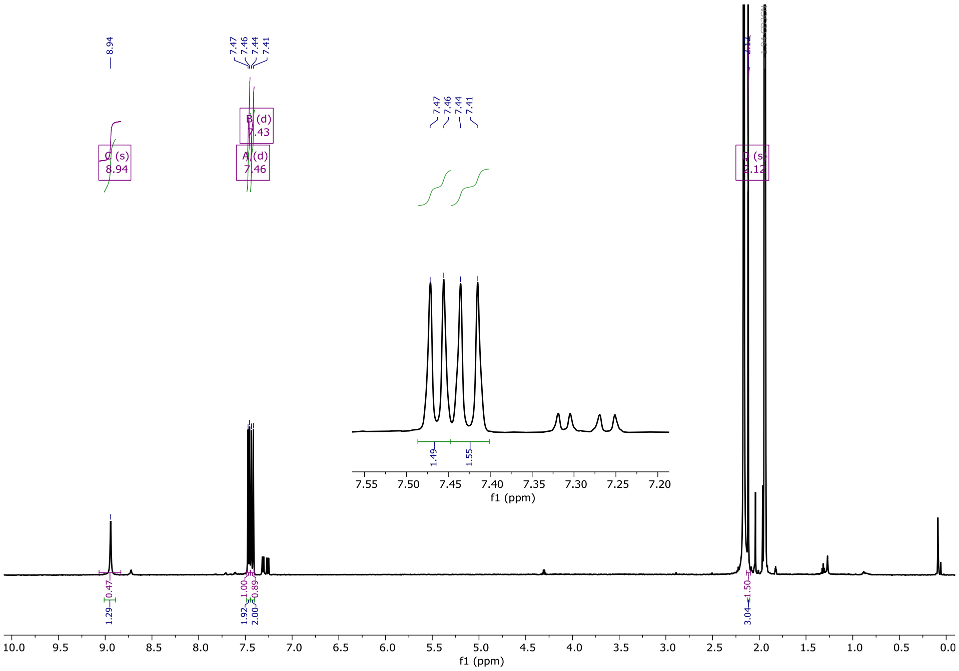

Figure S21. ^{1}H-NMR of compound **7** in CD_3CN with a drop D_2O.

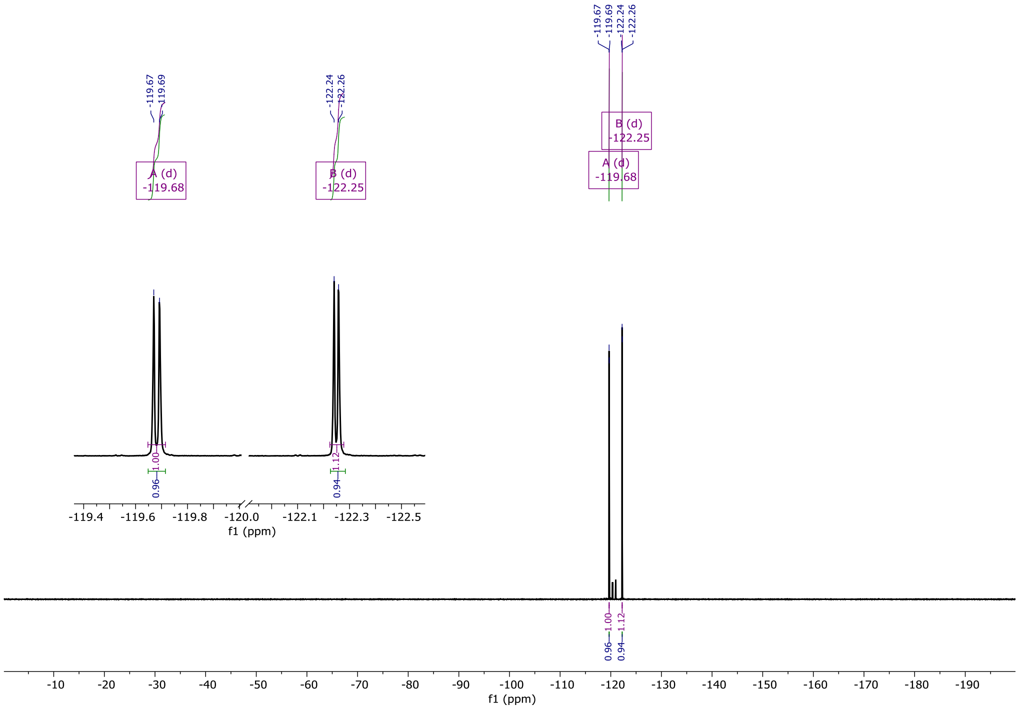

Figure S22. ^{19}F-NMR of compound **7** in CD_3CN with a drop D_2O.

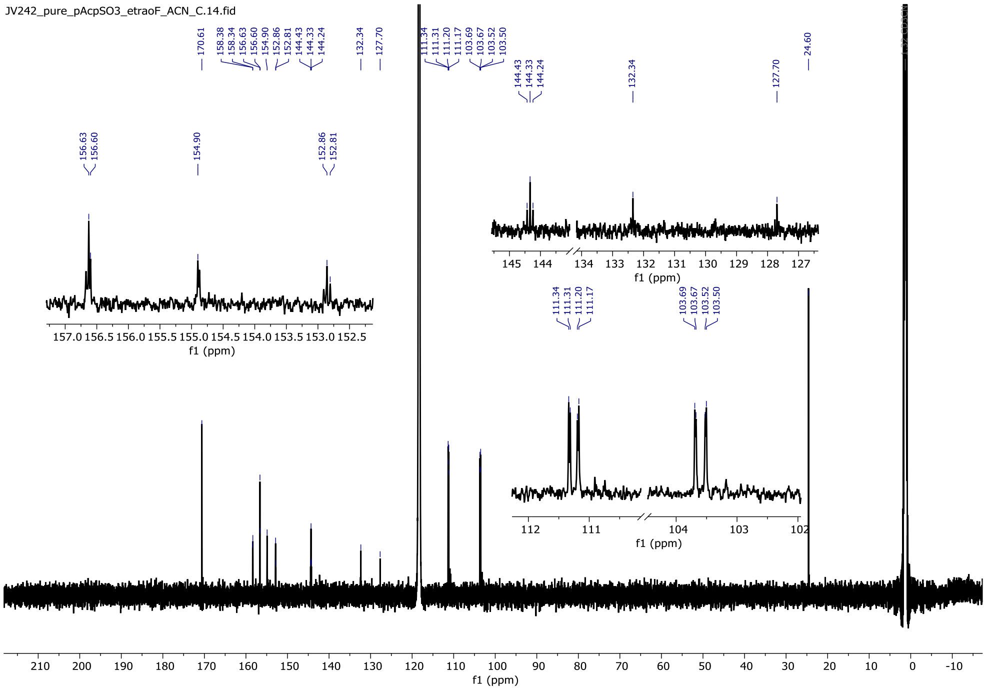

Figure S23. [1]C-NMR of compound **7** in CD_3CN with a drop D_2O.

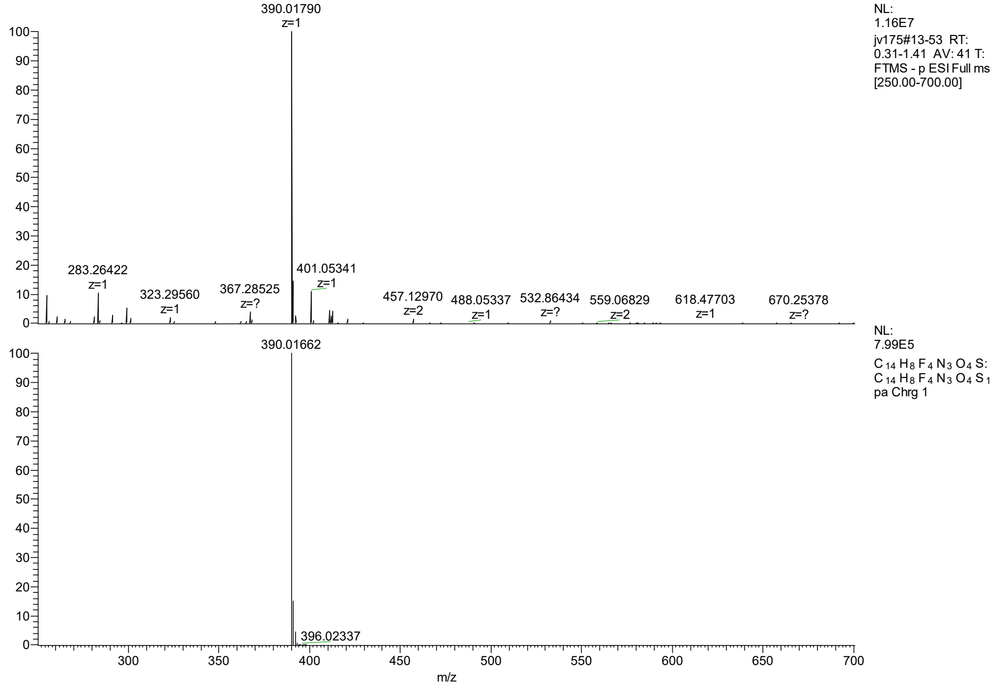

Figure S24. HRMS analysis of compound **7.**

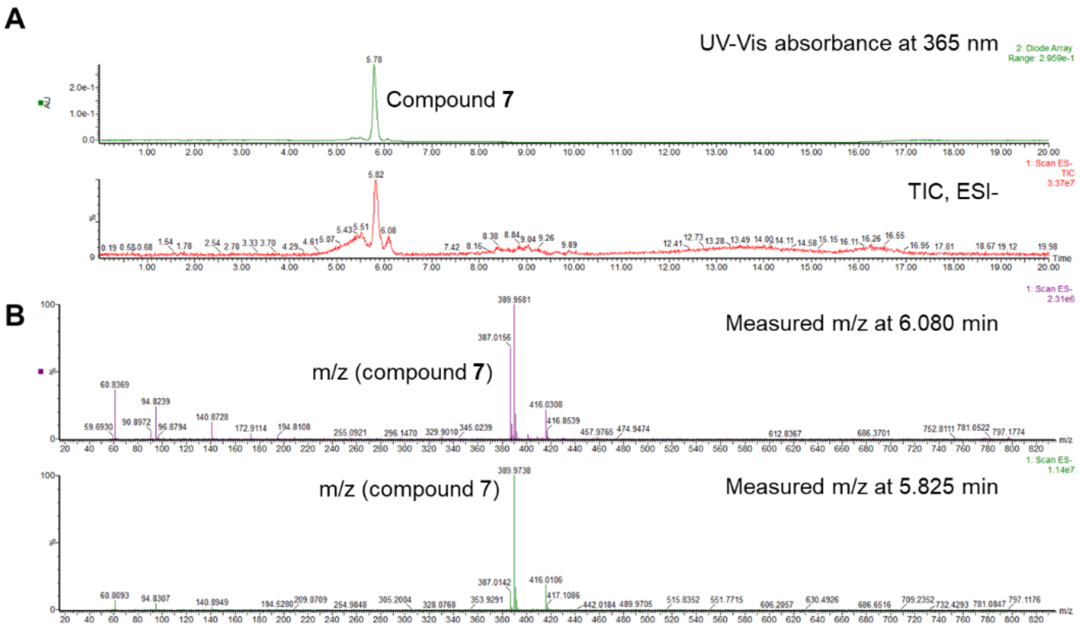

Figure S25. LCMS traces of isolated compound **7** measured in negative mode (Conditions: 0.1% NH_3 in water and 0.1% NH_3 in ACN as eluents, HSST3 C18 column). **A)** Measured absorbance at 365 nm and the total ion current (TIC) measured in negative mode. **B)** Measured m/z of observed peaks at noted retention times.

***trans*-4-((2-chloroacetamido-2,6-difluorophenyl)diazenyl)-3,5-difluorobenzenesulfonate (A)**

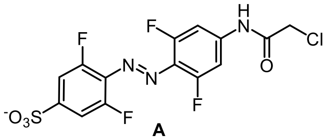

A

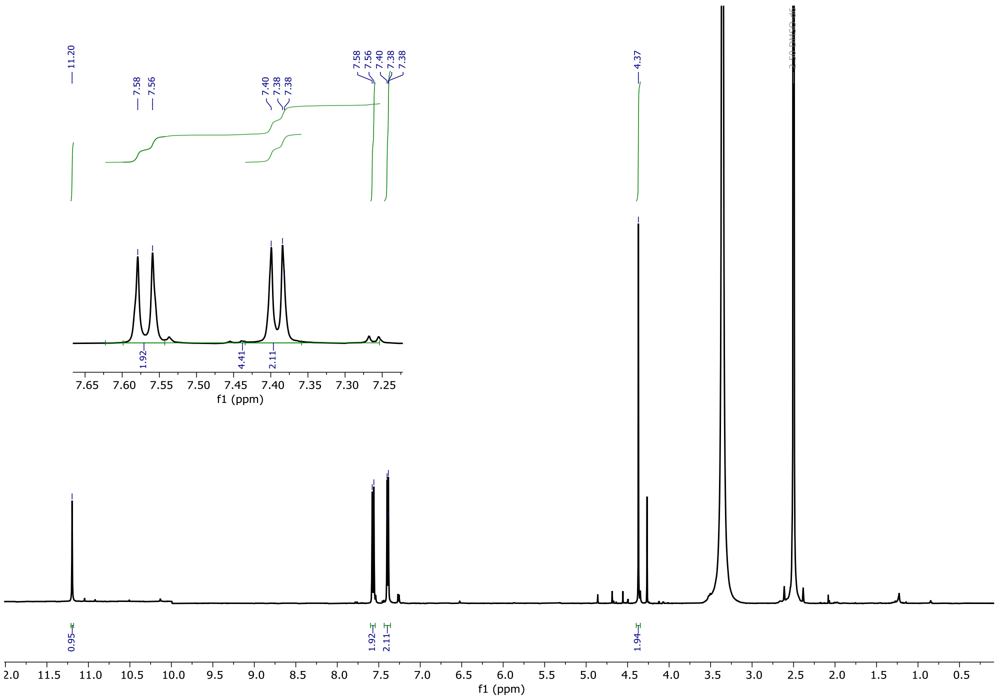

Figure S26. ^{1}H-NMR of compound **A** in DMSO-d_6.

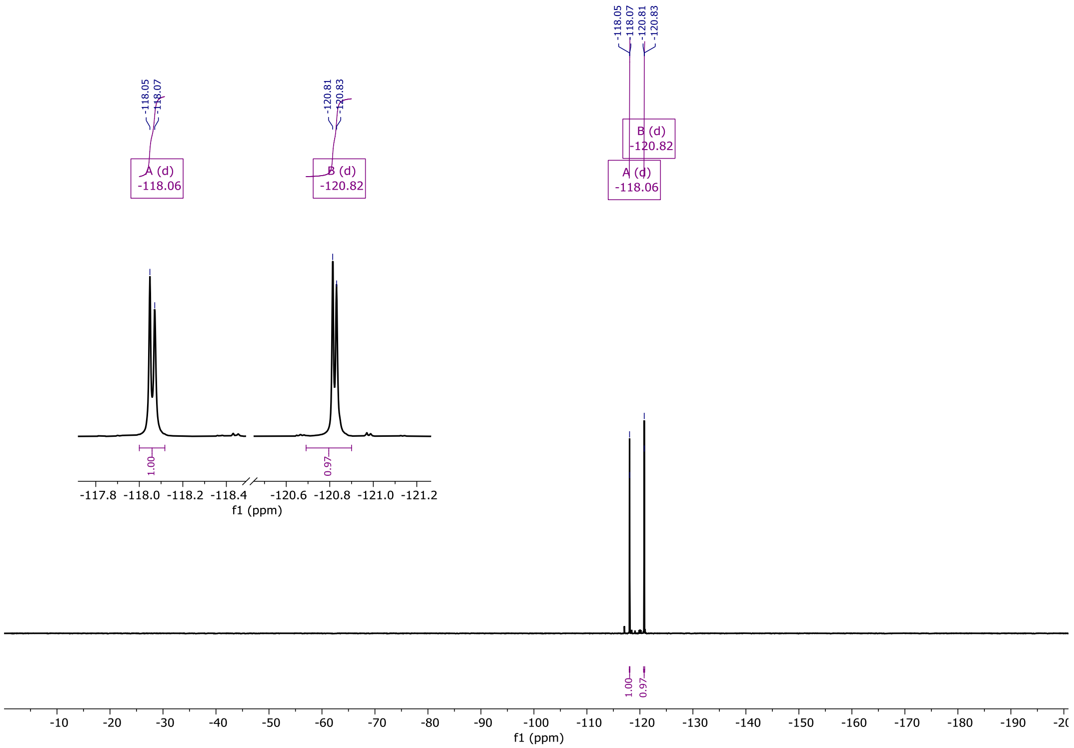

Figure S27. ^{19}F-NMR of compound **A** in DMSO-d_6.

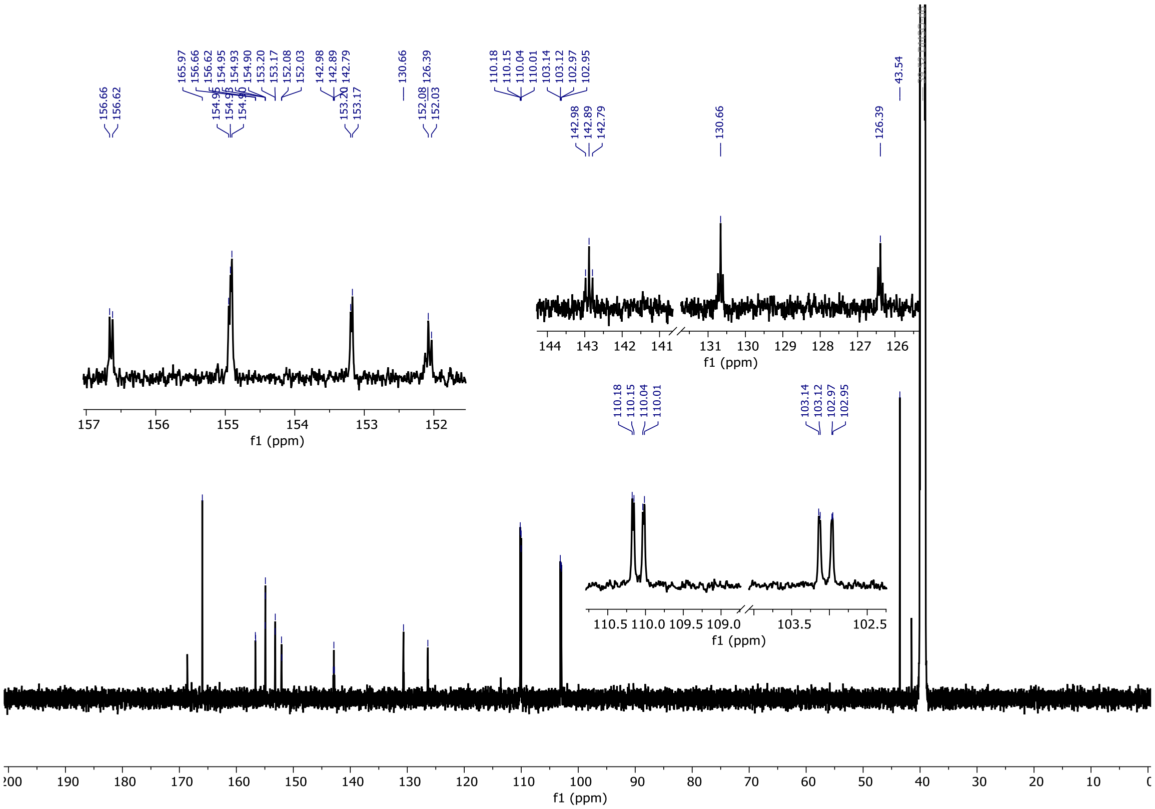

Figure S28. ^{13}C-NMR of compound **A** in DMSO-d_6.

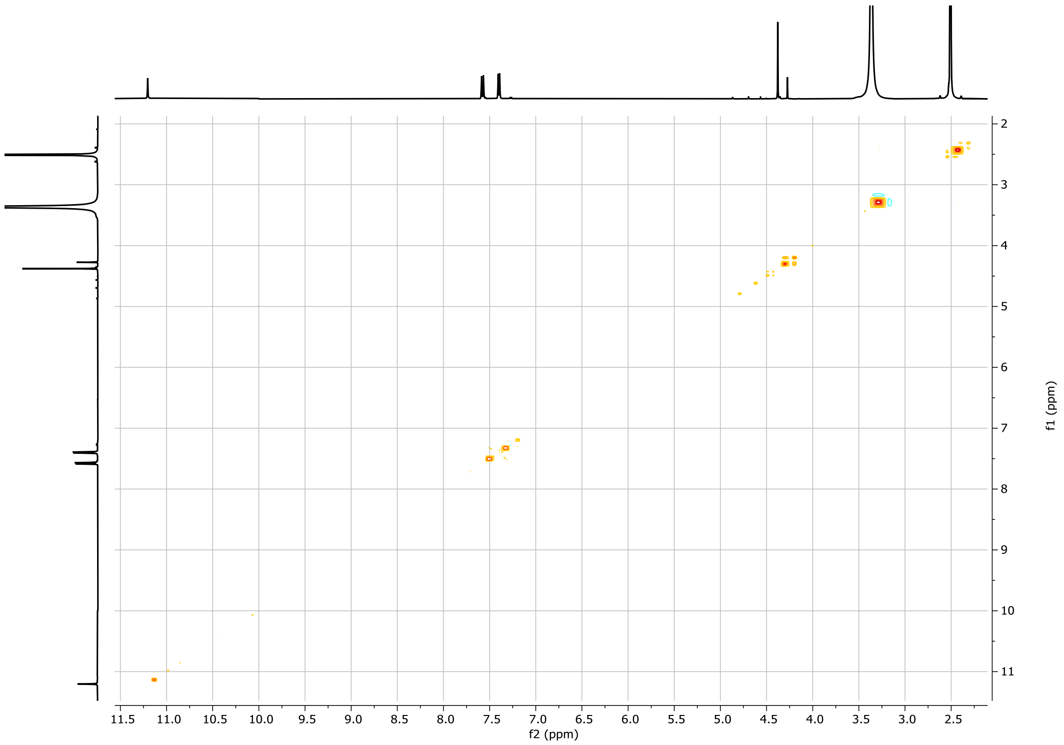

Figure S29. COSY 2D-NMR of compound **A** in DMSO-*d*6.

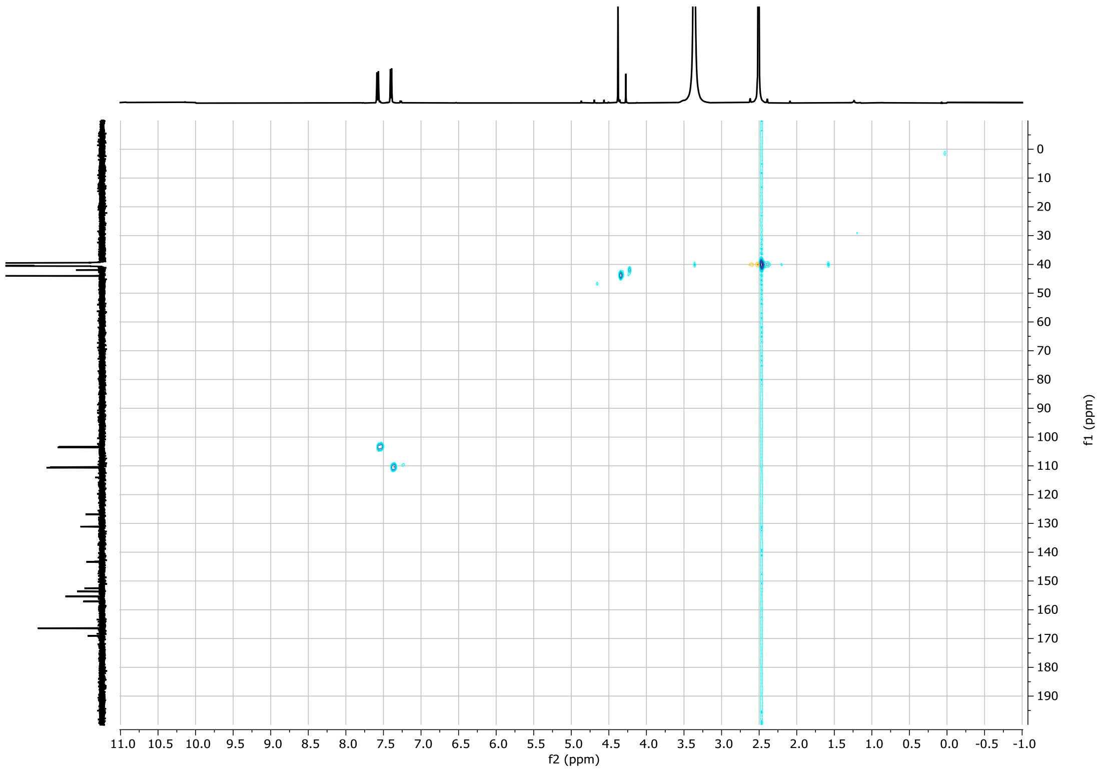

Figure S30. HSQC 2D-NMR of compound **A** in DMSO-*d*6.

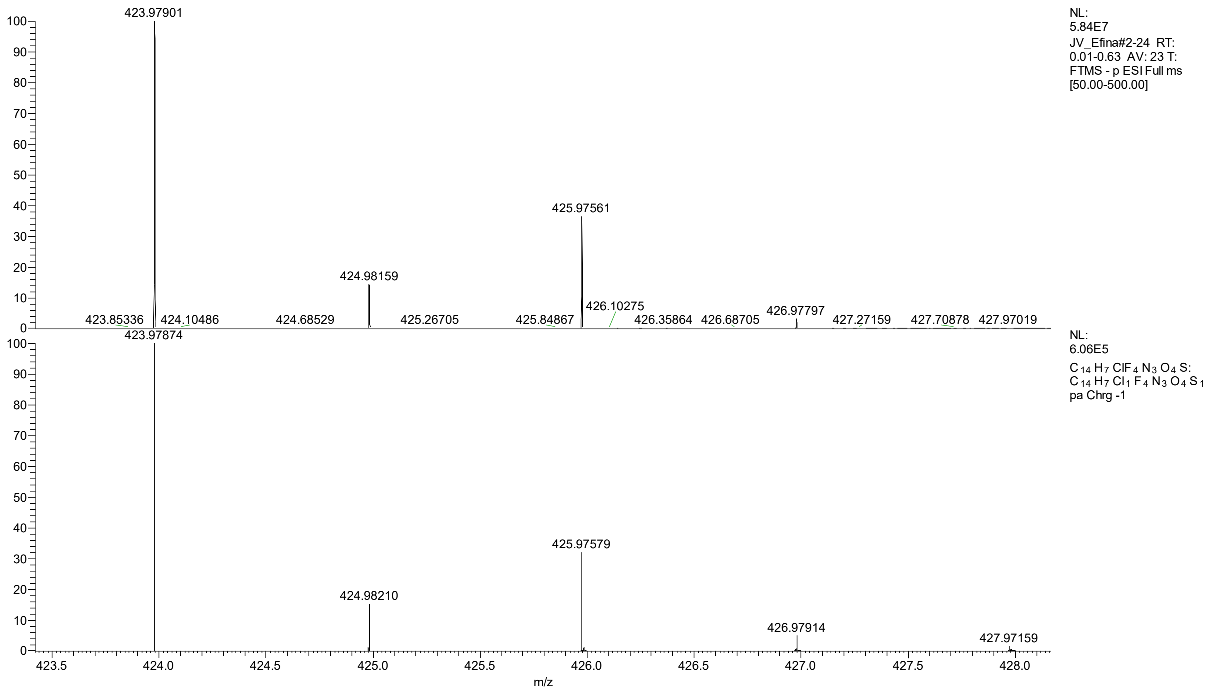

Figure S31. HRMS analysis of compound **A**$_6$.

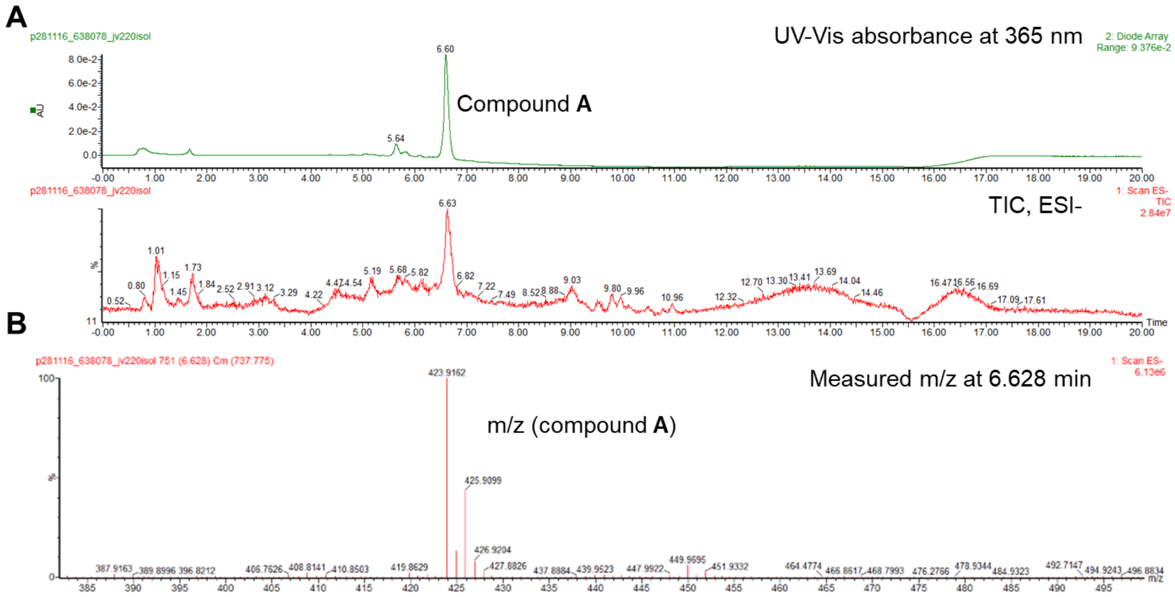

Figure S32. LCMS traces of isolated azobenzene **A** measured in negative mode (Conditions: 0.1% NH_3 in water and 0.1% NH_3 in ACN as eluents, HSST3 C18 column). **A**) Measured absorbance at 365 nm and the total ion current (TIC) measured in negative mode. **B)** Measured m/z of observed peaks at noted retention time.
